# FOR LOVE OR REWARD? CHARACTERISING PREFERENCES FOR GIVING TO PARENTS IN AN EXPERIMENTAL SETTING^*^

**DOI:** 10.1111/ecoj.12248

**Published:** 2015-10-07

**Authors:** Maria Porter, Abi Adams

**Affiliations:** Michigan State University and Oxford Institute of Population Ageing Oxford University and Institute for Fiscal Studies

## Abstract

Understanding the motivations behind intergenerational transfers is an important and active research area in economics. The existence and responsiveness of familial transfers have consequences for the design of intra and intergenerational redistributive programmes, particularly as such programmes may crowd out private transfers amongst altruistic family members. Yet, despite theoretical and empirical advances in this area, significant gaps in our knowledge remain. In this article, we advance the current literature by shedding light on both the motivation for providing intergenerational transfers, and on the nature of preferences for such giving behaviour, by using experimental techniques and revealed preference methods.

In this article, we are concerned with transfers made by adult children to their parents. It is clear that parents may invest in their children because they love them but also because of an expectation that their children will reciprocate to provide support for them in old age. However, there is no commitment mechanism available to parents to enforce that their children to provide the care that they expect.

So why do adult children provide support and resources to their parents in old age? This question is particularly salient in countries where parents have lower incomes than their children and rely on their adult children for financial support. It is also important for understanding what motivates grown children to devote time and other resources to ensure that parents in ill health receive the required care and support. More broadly, what motivates individuals to share scarce resources with family members? Early work addressing these questions determined that even selfish children could be incentivised to behave in the interest of the family by an altruistic patriarch (Becker, 1974).

Determining the primary motivation for familial transfers, specifically whether they are altruistically or strategically motivated, has long been a central question in the literature (Bernheim *et al.*, 1985; Cox, 1987) with consequences for a number of diverse areas in economics. For example, Ricardian equivalence is hard to obtain when children are altruistically motivated towards their parents (Bilbiie and Monacelli, 2013). However, it is difficult to disentangle the various motivations for intergenerational transfers in survey data. For example, while private transfers may decline when a recipient’s income increases, this does not necessarily mean that transfers are altruistically motivated, because other motives such as co-insurance cannot be ruled out (Kotlikoff and Spivak, 1981). Distinguishing between altruistic and strategic motives for giving is further complicated by the fact that there are many other reasons why people give: an aversion to unfairness or inequality (Fehr and Schmidt, 1999); the warm-glow of giving (Andreoni, 1989 1990); reciprocity – rewarding friendly actions or punishing hostile actions at a cost (Rabin, 1993; Camerer and Fehr, 2004); and reciprocal altruism – giving to generate or relieve an obligation (Camerer and Fehr, 2004; Cox *et al.*, 2004; Leider *et al.*, 2009; Ligon and Schechter, 2012).

Our main contribution to this broad literature is to uncover the characteristics of, and motivations for, giving between adult children and their parents, by using a carefully designed experiment. Subjects play a series of dictator games in the laboratory, once with parents and once with strangers as recipients, where the amount to divide and the relative price of giving vary across games. To our knowledge, Peters *et al.* (2004) is the only prior study to have examined behaviour between parents and children in the laboratory, although their study differs significantly from ours as they studied interactions between young children (aged 8–16) and their parents in a very different experimental setting.

Our experimental design enables us to explore the salience of reciprocal motivations for transfers between adult children and parents. The dictator game is generally used in experimental settings because reciprocation, either in the form of reward or punishment is not possible when the recipient is an anonymous stranger. However, we cannot maintain control of any subsequent interactions between subjects and parents outside the laboratory, and these interactions influence the behaviour we observe in the laboratory. Our experiments were designed with this in mind, and provide an example of how the line between ‘laboratory’ and ‘field’ can be blurred to gain some understanding of behaviour outside of the laboratory in a novel way.

To explore adult children’s motivations for giving, we vary the amount of information that parents receive about the games their children play in order to vary the likelihood of parental reciprocity. We find evidence of reciprocal motivations for sharing with parents, which differs from prior work using survey data that found evidence of altruistically linked family members (Altonji *et al.*, 1997). In our experiments, when participants were told that their parents would be receiving information about their choices, they gave more to their parents than those who were told their parents would not be informed of how payments were determined. If subjects had given to parents for purely altruistic reasons, then this information treatment would not have influenced the amount shared with them.

This novel experimental design also contributes to a strand of literature in experimental economics, which has shown evidence of reciprocal behaviour on the part of dictators in several different contexts (Hoffman *et al.*, 1996; Bohnet and Frey, 1999; Ben-Ner *et al.*, 2004; Cox *et al.*, 2004). In experiments in which recipients are friends, dictators share more with those to whom they are more closely connected (Goeree *et al.*, 2010). Similarly, dictators give more to close friends than to strangers, and these differences are strongest when the giving is not anonymous (Leider *et al.*, 2009). Our study differs from these two studies in three different dimensions.

First, in contrast to the latter two experimental studies, we show evidence for reciprocal motives for familial transfers without the confounding influence of selection effects. These past experimental studies on pro-social behaviour in social networks have found strong homophilous tendencies in choosing friends (Leider *et al.*, 2009; Goeree *et al.*, 2010). For example, people’s friends often exhibit similar levels of kindness, so that it is not possible to differentiate between the selection effect in choosing one’s friends from the social interaction effect (Leider *et al.*, 2009). We purposely designed our experiments to ensure that such a selection effect would not be possible. This is one reason why we required parents to be recipients, rather than a chosen family member. In order to ensure this, we asked participants to send payments to their mothers if both parents were alive but living separately from one another.

Second, these prior studies have not directly addressed the nature of preferences for giving within families, a setting in which further questions arise. In our article, we address these wider intrahousehold-specific questions to help inform, for example, recent work on the consequences of relaxing the assumption of perfectly transferable utility for explanations of the formation and dissolution of families (Giuliano, 2007; Chiappori *et al.*, 2012*a*,*b*).

Third, we conduct a more ambitious preference recovery exercise than Leider *et al.* (2009), which is in the spirit of Andreoni and Miller (2002), by collecting sufficient information on the choice behaviours of each subject. Using revealed preference and structural techniques, we use our experimental data to examine the rationality of intergenerational transfers, to recover how preferences for giving vary depending on the recipient of a gift and to examine the motivation for transfers from adult children to their parents. We find that the vast majority of subjects have consistent and well-behaved preferences for giving to strangers and parents when these transfers are treated as separate goods. We identify a series of preference ‘types’ in our subject pool and estimate the parameters of a Constant Elasticity of Substitution (CES) utility function. This allows us to examine the nature of preferences for giving and to explore how they vary by the recipient of a gift in great detail.

In doing so, we contribute to a second strand of literature in experimental economics. Our findings support the results of prior laboratory experiments with a similar experimental design in several different contexts: among young children (Harbaugh *et al.*, 2001; List and Millimet, 2008); among economics students and other adults (Sippel, 1997; Mattei, 2000; Andreoni and Miller, 2002); and with a broader set of budget constraints (Fisman *et al.*, 2007). In a further application of revealed preference methods, we go on to find that preferences for giving are conditional upon the recipient of a transfer. We find that when we pool the choices from the games with parents with those played with strangers, the choices of the majority of players violate axioms of revealed preferences. This indicates that most players view giving to parents and strangers as distinct goods, and they have different preferences for each one.

In summary, we find greater proclivity for giving and greater price sensitivity of transfers when parents rather than strangers are recipients of transfers. However, we uncover significant heterogeneity in preferences for giving to parents, which, to our knowledge, has not been explored in any previous work. Further, this is the first article to provide estimates of preference parameters for giving to parents on the part of adult children, which might be used to calibrate future macroeconomic multi-generation models. Finally, we find that many adult children do not share resources with parents in order to maximise social efficiency gains within the family. That is, a number of subjects do not exhibit preferences of perfect substitutes for giving to parents. For these subjects, the oft used assumption of transferable utility in modelling family behaviour may not be relevant.

The rest of this article is structured as follows. In Section 1, we describe our experimental design. In Section 2, we assess the rationality of subjects’ choices (to ensure that a consistent preference ordering can be found that rationalises their choices) and test whether giving to parents and giving to strangers can be treated as the same good in a subject’s utility function. In Section 3, we formally characterise the nature of preferences for giving to parents and strangers. In Section 4, we examine our subjects’ motives for giving to parents using the results of our controlled information experiment. Section 5 concludes.

## 1. Experimental Design

This Section describes our sample selection criteria and the design of our modified dictator games and information treatment.

### 1.1. Sample Selection

In recruiting subjects for our experiments, we focused upon adults who largely live independently from their parents. Further, we chose deliberately to exclude undergraduate students and those with a university qualification in economics from our study. Though undergraduate students live apart from parents, they often visit them, typically consider the parents’ address to be their permanent address, and they often rely on parents financially. Furthermore, student and non-student subjects, especially those with a background in economics, often show very different patterns of behaviour in laboratory experiments (Harrison and List, 2004).

As our experiments took place in Oxford, the majority of our sample resided in the southeast region of the UK. In comparing our sample to those in the British Household Panel Survey (BHPS) who reside in the southeast region of the UK, we over-sample women and those with a college degree. The extent to which our findings may be generalised to a wider population may reflect the extent to which gender and education may influence behaviour in this particular context, although our findings are robust to controlling for such characteristics. We refer the reader to the online Appendix B for further details of our recruitment procedures and subject pool.

### 1.2. Modified Dictator Game

We designed our experiment to test the rationality and characteristics of preferences for giving to parents and strangers. Each subject played a series of dictator games separately with a parent and with an unknown stranger, who was another subject chosen at random from those participating in the same session and whose identity remained anonymous. Rather than give a single amount to the subject to be divided up between himself and the recipient (as is usual in dictator games), each subject was tasked with allocating ‘tokens’ under a series of different budgets. Decision problems differed by the number of tokens to be divided and the amount of money that each token was worth. Tokens were worth 10, 20 or 30 pence. The total number of tokens varied between 40 and 100. Table 1 provides the details of the eleven budgets that the subjects faced.^1^ The order of the decision problems was randomised across subjects, and they were told that the experimenter would randomly choose one of the decision problems and carry it out.

Table 1 also details the average amount that our subjects chose to share from each of the budgets. In comparison to subjects in Andreoni and Miller (2002), our subjects were more sensitive to the relative price of giving, sharing 50% when the price of giving was less than one, 36% when the price was one and 30% when the price was greater than one. When we distinguish between games played with parents and strangers, we see that this sensitivity to price only holds for games played with parents. In games with strangers, our subjects were slightly more generous than those in Andreoni and Miller’s sample, giving 30% on average, irrespective of price. However, in their games with parents, our subjects gave about 70% of their share to parents when the relative price of giving was less than one, 45% when the price was one and 30% when the price was greater than one. These differences are statistically significant.

### 1.3. Information Experiment

As a further dimension to our experimental design, we randomised the amount of information parents received about the games played in the laboratory. This randomisation allows us to explore whether subjects are altruistically or strategically motivated to share with parents. Note that this randomisation occurred at the session level rather than the subject level to avoid confusion and potential spillovers. Subjects were not aware of these differences across sessions. All subjects in a session were assigned to one of three treatment groups:

1. Subject’s parent was notified that her child participated in a study, but no additional information was provided.
2. Subject’s parent was given full information regarding the dictator games that her child played with her, including complete instructions on the games, how the child played each game and how much was allocated to the parent and to the child.
3. Same as (2) above, but the subject was also given an opportunity to write a note to the parent that was included with the letter and payment mailed to the parent.

The third treatment group was implemented to give participants an opportunity to send their parents a message in case they were deterred from, for example, exhibiting perfect substitutes preferences out of concern that their parents might view this as selfish behaviour.^2^ If this were true, subjects could have been more likely to exhibit selfless or Leontief preferences in Treatments 2 or 3 because of concerns about their parents’ reaction to a small payment amount and a concern for being perceived of as fair (Andreoni and Bernheim, 2009).

Of the 64 subjects in Treatment 3, 41 wrote their parents a message. However, only four explained perfect substitutes behaviour. Four other subjects explained that they had tried their best to divide tokens so that total payouts were split equally. One subject explained selfish behaviour. The majority of those who wrote notes (32 subjects) did not send any message explaining their decisions in the game. For example, messages included: ‘Hi!’ and ‘Enjoy, Mum X’. All notes can be found in online Appendix A. The majority of subjects did not use the opportunity to write a note to their parents to explain behaviour, and we find there is little difference between Treatments 2 and 3 in affecting the amount shared with parents.

We also randomised whether subjects played first with their parents or with strangers and this randomisation was done across individual laboratory sessions. It is important to note that subjects were not provided with any details of the experiment in advance of their participation. Thus, if they played dictator games with strangers initially, they did not know that they would repeat the same games with parents. Likewise, if they played games with parents first, they did not know that this would be followed by another set of games played with strangers. This has important implications for how subjects would play, particularly with parents, and how they could have been influenced by the information treatment, which is discussed below.

Our 190 subjects were evenly distributed across the three treatment groups, with 66 subjects in Treatment 1, 60 in Treatment 2, and 64 in Treatment 3 (see Table 2). For those in Treatment 1, 37 subjects played with a stranger first and 29 played first with a parent. Of the 60 subjects in Treatment 2, 19 played with a stranger first and of the 64 subjects in Treatment 3, 33 played with a stranger first.

## 2. Are Preferences for Giving Rational?

We begin by examining whether choices are rational, that is whether some well-behaved preference ordering exists consistent with each individual’s choices in the laboratory. We do so by checking for violations of the Generalised Axiom of Revealed Preference (GARP) (Varian, 1982).^3^

We find that we can rationalise the behaviour of the overwhelming majority of our subjects by the standard utility maximisation model (see Table 3). About 91% of our sample satisfy GARP when playing with parents, while 89% of subjects satisfy GARP when playing with strangers. This difference is not statistically significant. These high pass rates are not the product of a weak test of rationality, as indicated by the measure of ‘predictive success’, *s* ∈ [−1,1] for our tests (Beatty and Crawford, 2011). This measure allows us to correct observed pass rates for the ‘demandingness’ of a revealed preference test, which is measured by the so-called ‘relative area’ *a*. An *s* in the neighbourhood of 1 indicates that the data satisfy strict restrictions (the ideal situation), whilst an *s* in the neighbourhood of −1, denotes the opposite; choice behaviour violating very weak restrictions.^4^

Age and education do not impact the likelihood of passing GARP. However, men are more likely than women to satisfy GARP, other things equal. Whereas 97% of men pass GARP in games with strangers, 87% of women do so. Similarly, 94% of men pass GARP with parents, and 86% of women pass GARP in games with parents. We refer the reader to online Appendix B for further details.

To determine whether preferences for giving depend on the recipient, we pool an individual’s choices from the games played with strangers with the games played with parents and check whether a well-behaved preference ordering exists that can rationalise this full choice set.^5^ We find that giving to parents and strangers cannot be rationalised by the same preference ordering for 73% of subjects (66% of men and 77% of women). For these individuals, giving to parents and strangers cannot be treated as a single good and preferences for giving are conditional upon the recipient. The greater ‘demandingness’ of the revealed preference test does not explain the significantly lower pass rate on the pooled choice set, as the predictive success measure is 0.266.

### 2.1. How Significant Are the Deviations From Rationality?

**Fig. 1:**
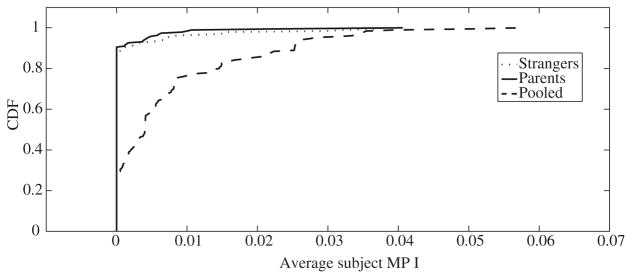
Empirical Cumulative Distribution Function of MPI

We compute the severity of the GARP violations to check whether behaviour is essentially rational and fails our test due to small random errors. We do so by computing the ‘money pump index’ (MPI) proposed by Echenique *et al.* (2011) for each subject. The MPI can be interpreted as the monetary value of tokens that could be extracted from a subject who behaves inconsistently. The severity of a GARP violation is then measured by the amount of money that a ‘devious arbitrager’ could have extracted from our subject. Money pump cost violations are relatively small when giving to parents and strangers are treated as separate goods, suggesting that choices are effectively rational (see Figure 1). However, when choice sets are pooled, GARP violations are much more severe, suggesting that preferences for giving are indeed conditional on the intended recipient.^6^

We also examined the number of budgets that had to be dropped for GARP violators to attain rationality. For most, only one budget had to be dropped. We did not find any patterns concerning the particular budget or timing of budget that had to be dropped. Further details are in online Appendix B.

## 3. Estimating Preferences for Giving

In this Section, we examine how preferences for giving differ by recipient. We do so by estimating preference parameters for giving to parents and strangers for those who satisfy GARP.

### 3.1. Preference Types

To characterise preferences for giving to parents and strangers, let *π_s_* represent payment to one’s self and *π_o_* represent the payment amount to the recipient, so that one’s utility is *u*(*π_s_*, *π_o_*). We group subjects into preference types depending on the similarity of their revealed preferences to four ‘extreme’ preference classes:

- perfectly selfish, *u*(*π_s_*, *π_o_*) = *u*(*π_s_*);
- perfect substitutes or utilitarian, *u*(*π_s_*, *π_o_*) = *π_s_* + *π_o_*;
- Leontief or Rawlsian, *u*(*π_s_*, *π_o_*) = min{*π_s_*, *π_o_*};
- perfectly selfless, *u*(*π_s_*, *π_o_*) = *u*(*π_o_*).

**Fig. 2:**
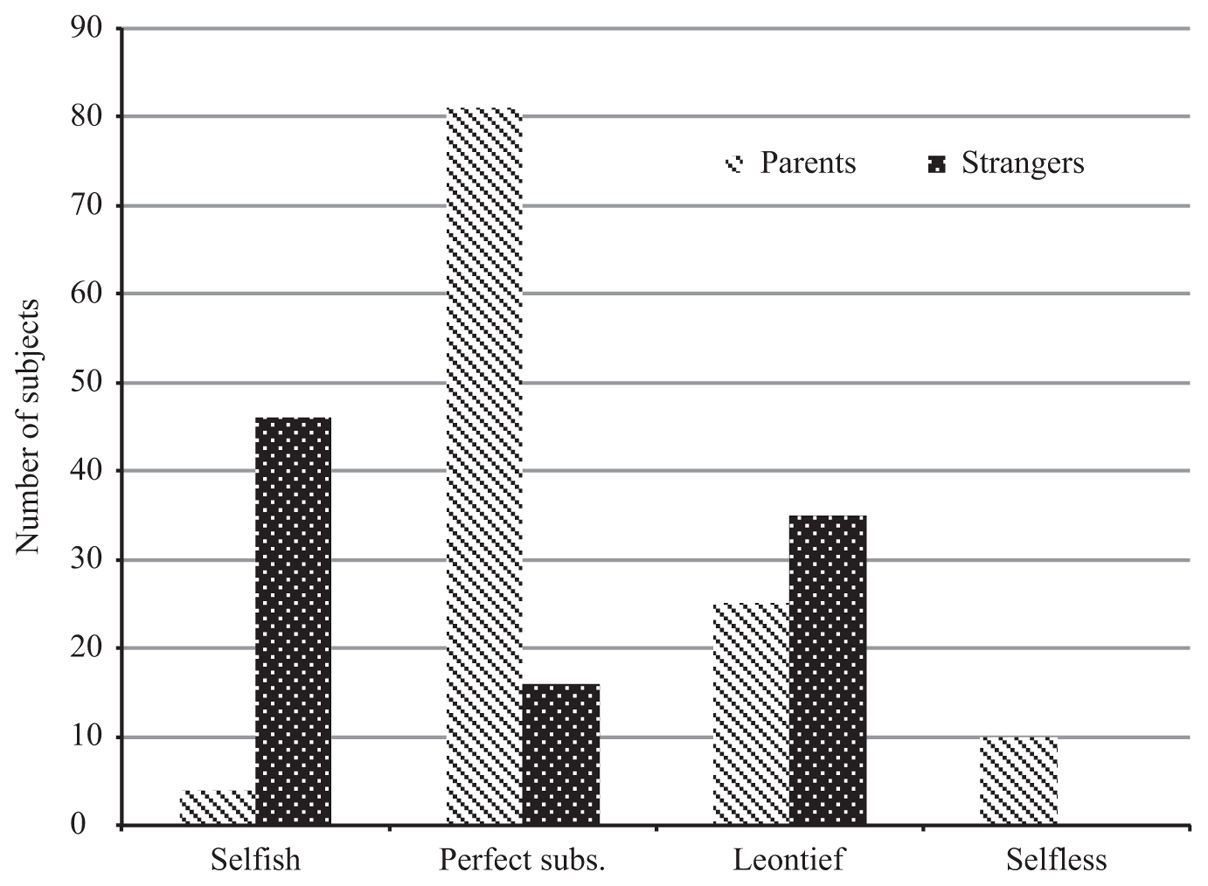
Distribution of Strong Preference Types

Many subjects’ choice behaviour can be perfectly rationalised by one of these ‘pure’ preference types: 59% with regard to their preferences over giving to strangers and 73% for parents. The distribution of preference types is significantly different across recipients (*χ*^2^ = 83.42) and displayed in Figure 2. Unsurprisingly, many more subjects played selfishly with strangers than with parents and pure selflessness occurred only with parents. In games with parents, the majority of subjects with strongly defined preferences exhibited a preference type of perfect substitutes, and thus acted to maximise joint payoffs. This finding of a higher proportion of perfect substitute types when giving to parents (which is statistically significant at the 1% level with a t-statistic of 16.8) also implies that giving to parents is more price sensitive than giving to strangers among those with strongly defined preferences. It is also interesting to note that an assumption of transferable utility between parents and children may be reasonable for those who play perfect substitutes with parents. However, as roughly half our sample, and over 30% of those with strong preferences, did not play perfect substitutes with their parents, our results cast some doubt on whether transferable utility is a valid assumption in general.^7^

There are 85 subjects whose preferences for both giving to parents and strangers are perfectly rationalised by one of the four preference categories. Table 4 gives the number of subjects with strong preferences that fall into each ‘parent – stranger preference’ cell. The three largest groups are:

- maximise family pay-offs – 32 subjects played selfishly with strangers but revealed perfect substitute preferences when playing with parents;
- equality in dictator-recipient pay-offs – 18 subjects split endowments equally, unconditional of recipient;
- maximise social pay-offs – 16 subjects revealed perfect substitute preferences irrespective of the identity of the recipient.

All subjects who played perfect substitutes with strangers, also did so with their parents, and thus comprise the latter. There are an additional eight subjects who played perfect substitutes with parents and Leontief with strangers; this group may have similar preferences to those who play Leontief with both recipients. The differences in games with parents may arise from differences in the extent to which players believe they can ‘undo’ the unequal shares in subsequent interactions with parents.^8^

### 3.2. Estimating Preferences

We classify subjects whose choices cannot be perfectly rationalised by one of the four preference types into ‘weak’ versions of these preference classes by assigning subjects the preference type that was ‘closest’ to their revealed preference. Specifically, we place subjects into the preference type with the minimal Euclidean distance between their actual choices and the choices dictated by the pure preference type.^9^ To get a more detailed picture of preferences within these weak types, we estimate preference parameters for a CES utility function within each weak preference type (with the exception of the ‘weakly selfless’ category due to limited observations).^10^ The functional form of the CES utility function is:

(1) $$u(\pi_{s},\pi_{o})={\left[a\pi_{s}^{\rho }+(1-a)\pi_{o}^{\rho }\right]}^{1/\rho }.$$

The parameters have clear interpretations: *a* gives the weight on ‘own’ consumption, indicating the degree of selfishness (*a* = 1 when perfectly selfish and *a* = 0 when perfectly selfless), while *ρ* determines the elasticity of substitution, *σ* = 1/(*ρ* − 1), between one’s own payoff and that of the recipient. As *ρ* approaches −*∞*, preferences are Leontief. When *ρ* = 1, preferences are perfect substitutes. With the budget constraint *π_s_* + *pπ_o_* = *m*, the CES demand function is:

(2) $$\begin{array}{l} \pi_{s}(p,m)=\frac{{\left[a/(1-a)\right]}^{1/(1-\rho )}}{p^{-\rho /(\rho -1)}+{\left[a/(1-a)\right]}^{1/(1-\rho )}}m\\ =\frac{A}{p^{r}+A}m, \end{array}$$

where *A* = [*a*/(1 − *a*)]^1/(1−^*^ρ^*^)^ and *r* = −*ρ*/(*ρ* − 1). *A* and *r* are estimated using a two-limit non-linear tobit by maximum-likelihood to take into account the fact that subjects’ choices are censored at both ends of the budget constraint. To remove heteroscedasticity in the error term in levels, demands are estimated as budget shares with an i.i.d error term. The estimated demand function is then:

(3) $$\frac{\pi_{s}(p,m)}{m}=\frac{A}{p^{r}+A}+\varepsilon ,$$

where *ε*^~^N(0, *σ*^2^). Table 5 gives our results. We find a greater proclivity to give to parents and some evidence that giving to parents is more price responsive. *a* is highest amongst those with weakly selfish preferences and, as we might expect, *a* is higher when strangers as opposed to parents are recipients.^11^ There is considerable variation in estimated *ρ* within our sample. For those with weakly Leontief preferences, the estimated *ρ* is statistically significant, negative and relatively high in magnitude (in line with what we would expect). For those in the weakly perfect substitutes category, we find that the marginal rate of substitution between own and recipient pay-off is greater when playing with parents, and that this difference is statistically significant, suggesting greater price responsiveness when giving to parents for this group.

## 4. Information Experiment

In this Section, we explore the motivations for transfers to parents. We differentiate between whether subjects give to parents because of altruism – either pure altruism or altruistic reciprocity (reciprocating kindness shown previously by their parents), or because of some reciprocal or strategic motive. If adult children are altruistically motivated, their preferences over payments to parents relative to payments to themselves should not differ by treatment group. But if adult children are strategically motivated to share with parents, then they would value giving to parents quite differently depending on the degree to which parents are informed of their decisions in the laboratory. Parents who receive full information may be inclined to either reward generosity and perhaps share the winnings of the experiment, or to punish a child’s selfishness and perhaps reduce subsequent transfers to the grown child.

An alternative to the latter explanation of strategic motives for giving is that parents may derive a ‘signal value’ from a child’s gift, which is stronger when they have more information. For example, parents may feel more loved if they see that their grown child has sacrificed tokens in order to share more with them. However, we do not believe that signalling is a compelling interpretation of our results. Subjects in Treatment 3 had an opportunity to write a note to their parents in which they could have provided some signal of love and explained that any possible zero payments were due to the fact that they maximised joint pay-offs. Yet, a very small number of subjects indicated the latter, and none provided a signal of love. Rather, those who played perfect substitutes indicated the possibility of undoing the experiment later on. In addition, we find no differences in preferences or payments between Treatments 2 and 3. We also observe interesting differences in behaviour by treatment group depending on whether a subject played first with strangers or first with parents that is difficult to nest within a signalling narrative.^12^

Finally, we note that we assume that it would have been prohibitively costly for participants in Treatment 1 to explain the details of the study fully and credibly to parents given that the study is relatively complicated to explain and would have required a long conversation with a parent. We made no contact with subjects or parents after the experiment to determine if this was the case. This was to assure participants’ privacy in their decisions in the laboratory.^13^

### 4.1. How Information to Parents Affects Preference Type

We find that the information treatment affects preferences towards giving to parents depending on one’s preferences towards giving to strangers. Table 6 records the differences between the ‘full information’ and ‘no information’ treatment groups in the proportion who have strong preferences of type *j* when giving to strangers, who then have strong preferences of type *i* when giving to parents. This change is calculated as follows:

(4) $$p_{ij}=\frac{n_{ij}^{\mathrm{FullInfo}}}{\sum_{i}{n_{ij}}^{\mathrm{FullInfo}}}-\frac{n_{ij}^{No\;\mathrm{Info}}}{\sum_{i}{n_{ij}}^{No\;\mathrm{Info}}},$$

where *n_ij_* is the number of subjects with preferences to parents in category *i* and preferences to strangers in category *j*.

Players who are perfectly selfish towards strangers are significantly less likely to behave selfishly towards parents who are informed about details of the game, and they are more likely to share pay-offs equally with them. This is strong evidence that players with generally selfish preferences for giving may be strategically motivated when giving to parents. It is only when parents are informed about the game that they may want to appear equitable to parents who may reciprocate after the game.

Interestingly, among those who reveal a preference for equity with strangers, subjects in the information treatment are less likely to play Leontief with parents and more likely to play perfect substitutes. Thus, subjects with a preference for sharing equally are more likely to maximise pay-offs when parents are more likely to share their winnings post-game. However, this difference is not statistically significant, perhaps due to the small sample size.

### 4.2. The Effect of Information to Parents on CES Parameters and Gift Amounts

For those with weakly categorised preferences, we estimate the parameters of a CES utility function as previously. However, due to sample size limitations, we estimate parameters within each treatment cell, pooling the observations of subjects with weakly categorised preferences within these groups. We find the weight on own consumption is statistically significantly lower amongst those in the full information treatment group, which is again suggestive of strategic motives for giving to parents amongst those with weak preferences (Table 7).

We use regression analysis to examine the marginal effect of the information treatment on payments to parents, where the dependent variable is the payment amount to the recipient in each game and the unit of observation is the game rather than the subject. Standard errors are clustered by respondent. Table 8 summarises the results. We find that subjects exposed to Treatments 2 and 3 give larger payments to parents, all else equal. These coefficient estimates indicate an average increase in giving to parents of about 50%, as the average value of tokens passed to parents was £5.^14^ In addition, the relative price of giving is a significant factor in determining payment amounts to both recipients; gifts to both recipients are normal goods.^15^ These results are also robust to controlling for individual characteristics: gender; age; education; student status; number of children of one’s own; number of biological children; and parents’ living arrangements, to control for whether the payment recipient is both parents, father only or mother only.

**Fig. 3:**
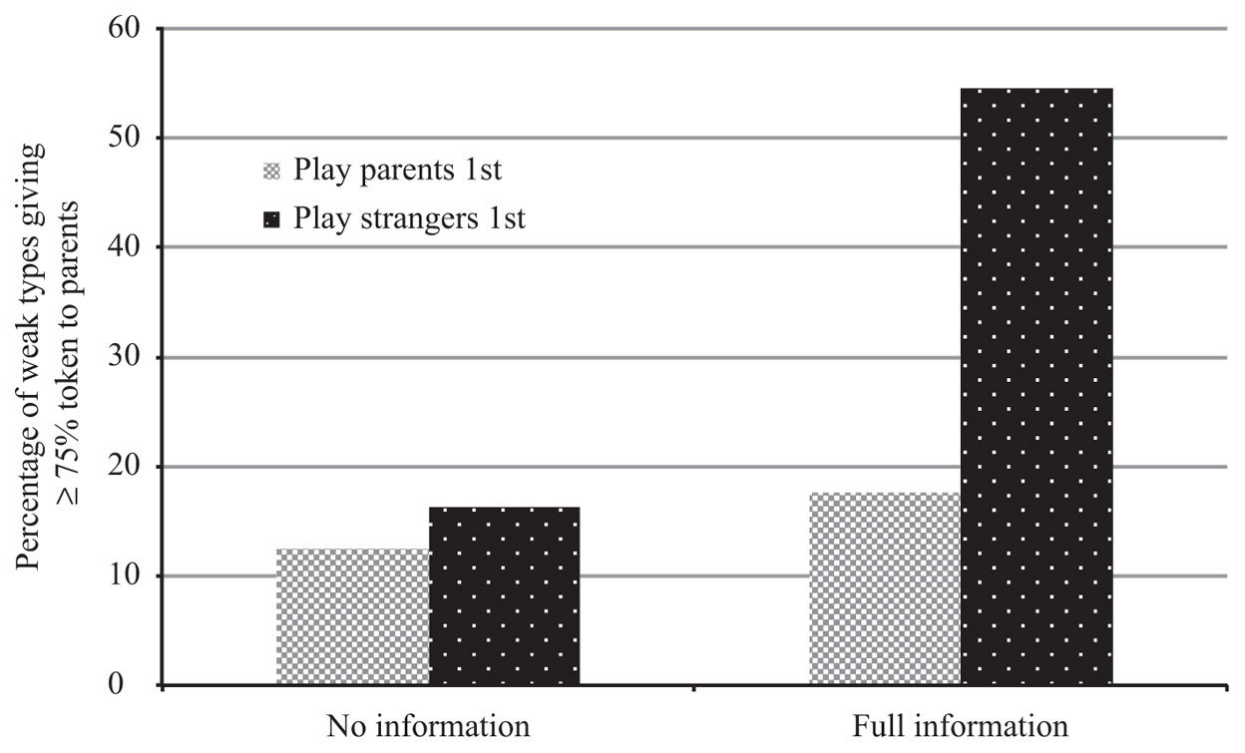
Percentage of Budgets at which Players Shared Over 75% of Tokens with Their Parents

Interestingly, when we separate the sample by those who played with strangers first and those who played with parents first, the treatment effect only holds for the sample of players who initially played with strangers. Note that subjects did not know any details of the experiments in advance. Thus, when playing with strangers initially, subjects did not know they would then play the same games with parents and *vice versa*. In addition, when subjects in the full information treatment had played with strangers first, a large proportion of their endowment of tokens was given to parents; in 55% of budgets played with parents after playing with strangers, subjects with weak preferences gave away at least 75% of their tokens to parents. However, among subjects who played the dictator games with parents first, there was a much smaller difference across information treatments in the likelihood to give a large proportion of one’s endowment to one’s parents at any particular budget (see Figure 3).

### 4.3. Theoretical Rationalisation of the Effect of Recipient Order

Our results are suggestive of strategic motives for transfers between adult children and their parents. However, we have found that the effect of the information treatment is much stronger when subjects with weak preferences have played with a stranger before playing with one’s parent.^16^

One might think that the differences found in playing with strangers initially might be explained by a learning effect. However, if there were a learning effect, then we would see a similar pattern for those who play with strangers first, regardless of the information treatment. Yet, subjects give more to parents only in the case of full information to parents and when playing with strangers first.

Alternatively, one might consider that games with strangers provide a reference point for subsequent games with parents, the idea being that if a player plays with strangers before playing with parents, then that player may give more to parents than the amount given to strangers, where the latter would serve as a reference point in games with parents. However, again, if such differences were to influence subsequent games with parents in the latter case, then we would not observe such large differences across information treatments.

Rather, we explain this empirical finding as an income effect in the presence of strategic motives. When a subject first plays with strangers, they come to the round of dictator games with parents with some extra amount of lump sum income from their winnings in the first set of games.^17^ We would expect this additional income to boost gifts to parents, given that our regression results indicate that gifts to parents are a normal good for those with weak preferences (see Table 8).

We could have avoided this income effect if we had chosen to pay subjects for one decision from all 22 budgets, rather than paying them for one in each of the 11 decisions with the different recipients. We chose to pay subjects as we did because we did not want subjects to be aware of the second game when playing the first game. Subjects received no advance information regarding the experiment, so that those who played with parents or strangers first had no reason to expect that they would subsequently play the same set of games with strangers or parents. If we had not done this, we would not have had a clean way to determine how the two games impacted each other. Future work might explore the impact of alternative payment mechanisms.

We hypothesise that the positive income effect, combined with the presence of strategic motives for giving to parents, provides a compelling explanation for our results. The CES estimates suggest that subjects in the full information group place higher weight on their parents’ pay-off, which is what we would expect from those with strategic motives for giving to their parents. The additional pay-offs from games with strangers then act to magnify the impact of this variation in preferences.

**Fig. 4:**
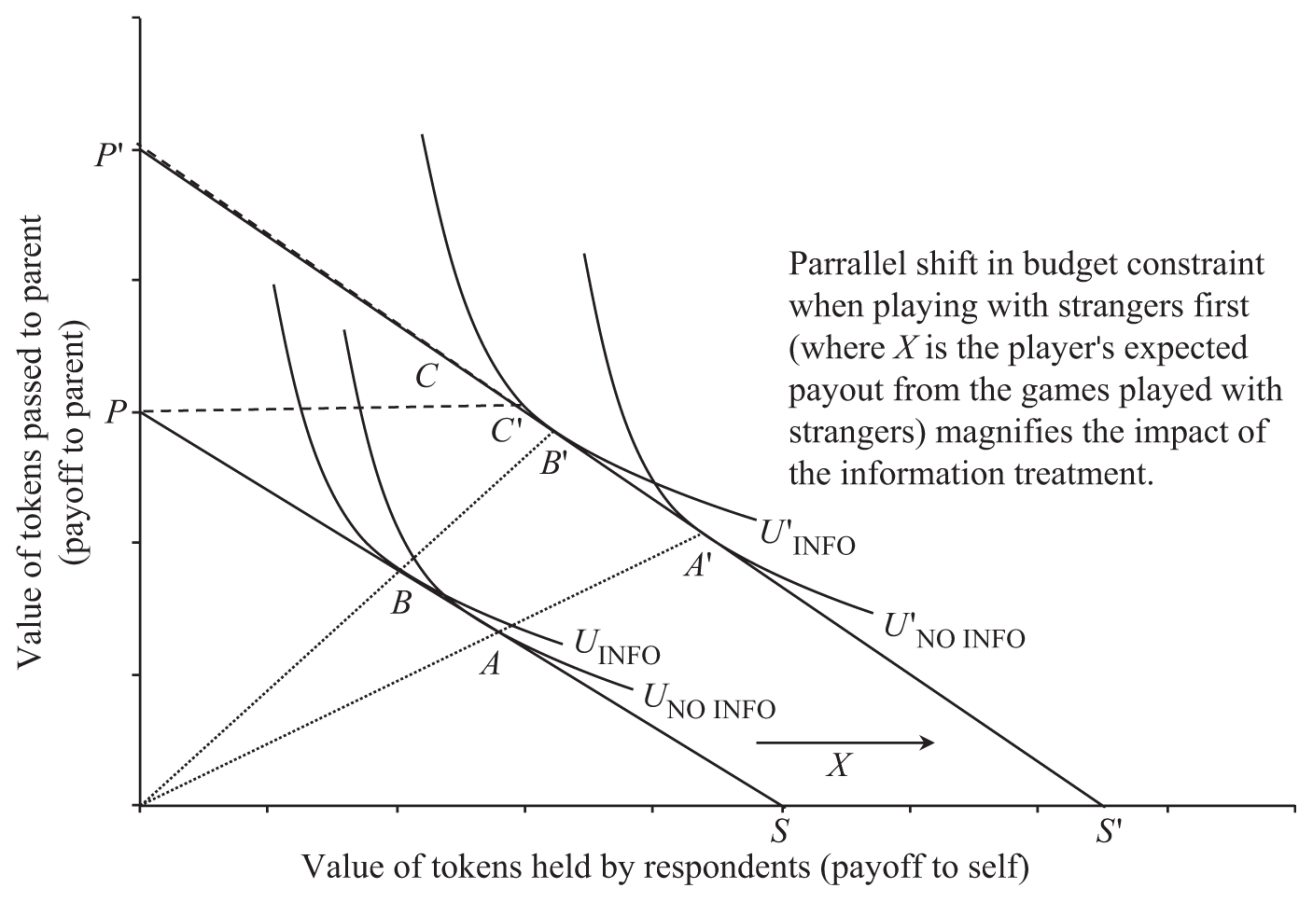
Strategic Motives and the Income Effect

To illustrate this argument, consider Figure 4, which depicts a hypothetical dictator game with parents. When a subject plays initially with parents rather than with strangers, she faces budget constraint *SP*. A subject playing with parents can keep all of the tokens, earning a pay-off of *S*, can give all of the tokens to parents, earning them a pay-off of *P* or can choose any allocation along the budget constraint *SP*. As those in the full information group place greater weight on parents’ payments than those in the no information group, the latter will choose allocation *A*, whereas the former will choose allocation *B*. Since our participants did not play selflessly with strangers, subjects who initially played with strangers played subsequent games with parents with some positive amount expected from these prior rounds. This causes a parallel outward shift of this budget constraint by some positive amount *X*.^18^ A subject would then be faced with budget constraint *S′P′*. However, not all points on this budget constraint are possible; any allocation along the budget constraint *S′P′* that is above *P* (e.g. *C*) is not directly available, as the subject does not have a mechanism by which he can trade-off her additional payment *X* for increased payment to parents in the experimental setting (this region is indicated by a dashed line). For simplicity, imagine that subjects are endowed with homothetic preferences (in line with our choice of CES utility function). We can see that those in the full information group, who place higher weight on their parents’ consumption, will choose a point *B′* over *A′*, sharing very generously with parents. The monetary divergence in pay-offs between treatment groups is also larger between *A′* and *B′* than between *A* and *B*. In addition, subjects who play strangers first and are in the full information group are more likely to be rationed over the total amount of tokens that they can pass to their parents (choosing *C′* as *C* is unavailable). Allocations *B′* and *C′* would explain the bunching of very large transfers to parents shown in Figure 3.

## 5. Conclusion

In this article, we make use of a novel experimental design to recover the characteristics of, and motivations for, giving to parents by adult children. We find that when parents rather than strangers are recipients of transfers, respondents have a greater proclivity for giving and greater price sensitivity for transfers. The latter would suggest that reducing the transaction costs of giving to parents may result in social efficiency gains. However, it is important to note that we uncover significant heterogeneity in preferences for giving to parents, which, to our knowledge, has not been explored in any previous work. Such heterogeneity in preference parameters for sharing resources across generations may need to be considered in multi-generational models of consumption and investment, which typically assume either perfectly altruistic or perfectly selfish overlapping generations.

Further, we find evidence of adult children being strategically motivated to share with parents. For those with strongly defined preferences, those who played selfishly with strangers also did so with parents who had no information but they shared equally with fully informed parents. In addition, for those with weakly defined preferences, we estimate a lower weight on own pay-off and a greater likelihood of sharing a large proportion of one’s budget when parents received information about the experiment. This evidence suggests that our subjects are strategically motivated when sharing with parents, as they share more with parents who are more likely to reciprocate in subsequent interactions. However, it is the subjects who initially play dictator games with strangers who are particularly affected by this change in information to parents. We hypothesise that this is because of an income effect influencing those who initially play with strangers.

These findings provide an important contribution to the literature on intergenerational transfer motives, as it is the first experimental study to examine motives for giving between parents and adult children. By having adult children play dictator games with a designated family member, we show evidence of reciprocal behaviour that is not due to a selection effect. We also show that while our subjects pass GARP, many of them do not behave in a way that would be consistent with the assumption of transferable utility that is often critical to many household models.

It would be interesting to use these methods to explore such motivations in developing countries, where elderly parents rely more on children than on public transfers for financial support, and where financial transfers generally flow from adult children to parents (whereas in the UK and other industrialised countries, financial transfers flow from parents to children and elderly parents rely on own savings or public support). China may be one particularly interesting and relevant case, as the one-child policy has meant that many adults are responsible for supporting four parents without any siblings to help them. There has been some evidence of crowding out of public transfers in developing countries (Cox *et al.*, 2004) but by less than what would be predicted under a model of altruism (Cox and Jimenez, 1992). Experimental work with migrants has found that remittances may be strategically motivated (Ambler, 2012), though the majority of recipients in this study were not close family members (spouses, parents, children), and it would be interesting to examine whether migrants behave similarly in this case.

Future work using a combination of laboratory experiments and survey data may shed more light on these areas. While the laboratory is restricted to monetary exchange, preferences for giving to parents can also be exhibited in other ways outside the laboratory. For example, adult children may provide time rather than money to parents (Levitt and List, 2007). Future studies using these methods might also employ additional variations within subjects. For example, giving to parents could be compared to giving to charities. Finally, as there is a great degree of heterogeneity in sharing in the laboratory, it would be interesting to explore what individual characteristics or factors outside the laboratory (e.g. number of siblings, gender, frequency of contact with parents) might influence such variation.

## Supplementary Material

appendix **Appendix A.** Subjects’ Notes.
**Appendix B.** Supplementary Information on Experiments and Related Analysis.
**Appendix C.** Laboratory Materials: Instructions to Subjects and Letter to Parents.
Data S1.

## Figures and Tables

**Table 1:** Budget Characteristics and Allocation Decisions

Total tokens	Hold value	Pass value	Price of giving	Average ‘give’ budget share			
				All	Stranger	Parents	Difference
40	10	40	0.25	0.50	0.29	0.70	−0.41***
40	10	30	0.33	0.50	0.29	0.71	−0.42***
60	10	20	0.50	0.51	0.30	0.72	−0.42***
75	10	20	0.50	0.51	0.29	0.72	−0.42***
60	10	10	1.00	0.36	0.26	0.45	−0.19***
80	10	10	1.00	0.36	0.26	0.45	−0.18***
100	10	10	1.00	0.36	0.27	0.45	−0.18***
60	20	10	2.00	0.29	0.28	0.31	−0.03
75	20	10	2.00	0.29	0.28	0.31	−0.03
40	30	10	3.00	0.31	0.30	0.32	−0.02
40	40	10	4.00	0.31	0.30	0.32	−0.02

Notes.

*** Statistically significant at the 1% level;

** Statistically significant at the 5% level;

* Statistically significant at the 10% level.

**Table 2:** Number of Observations By Treatment Group

Treatment	Play strangers 1st	Play parents 1st	Total
T1. No information	37	29	66
T2. Full information, no notes	19	41	60
T3. Full information, with notes	33	31	64
Full information (T2 and T3)	52	72	124
Total	89	101	190

**Table 3:** GARP Pass Rates

Giving to parents and strangers treated as separate goods			
	Pass rate	Number observations	Predictive success (*s*)
Giving to parents	0.905 (0.022)	172	0.854 (0.022)
Giving to strangers	0.884 (0.024)	168	0.833 (0.024)
Difference	0.021 (0.016)	4	0.021 (0.016)
Giving to parents and strangers treated as the same good			
Giving	0.268 (0.023)	51	0.266 (0.023)

*Note.* Standard errors in parentheses.

**Table 4:** Preference Types (Number of Subjects)

		Stranger as recipient			
		Selfish	Perfect substitutes	Leontief	Selfless
Parent as recipient	Selfish	4	0	0	0
	Perfect substitutes	32	16	8	0
	Leontief	4	0	18	0
	Selfless	0	0	7	0

**Table 5:** CES Weak Preference Parameters

	Selfish			Perfect substitutes			Leontief		
	Parents	Strangers	Difference	Parents	Strangers	Difference	Parents	Strangers	Difference
*A*	5.937 (0.155)	7.452*** (0.097)	−1.515*** (0.183)	0.996*** (0.048)	1.462*** (0.083)	−0.466*** (0.096)	0.993*** (0.021)	1201*** (0.021)	−0.208*** (0.030)
*r*	0.198* (0.185)	0.173 (0.114)	0.026 (0.217)	−0.900*** (0.083)	−0.520*** (0.107)	−0.381*** (0.135)	0.781*** (0.025)	0.773*** (0.024)	0.007 (0.035)
*a*	0.902***	0.919***	−0.017	0.500***	0.562***	−0.063***	0.493***	0.692***	−0.200***
***ρ***	−0.248*	−0.209	−0.039	0.474***	0.342***	0.132***	−3.560***	−3.415***	−0.144
*σ*	0.802*	0.828	−0.026	1.900***	1.520***	0.381***	0.219***	0.227***	−0.007
**ln (L)**	−44.80	−45.11		−104.80	−36.82		21.78	32.50	
*n*	44	165		165	187		220	330	

*Notes.* Standard errors in parentheses.

*** p < 0.01,

** p < 0.05,

* p < 0.1.

**Table 6:** Preference Switching Across Information Treatments (p_ij_)

		Stranger as recipient		
		Selfish	Perfect subtitute	Leontief
Parent as recipient	Selfish	−13.33*	0.00	0.00
	Perfect sub.	0.00	0.00	4.89
	Leontief	13.33*	0.00	−4.51
	Selfless	0.00	0.00	−0.38

Note.

*** p < 0.001,

** p < 0.05,

* p < 0.1.

**Table 7:** CES Parameters by Information Type

	No information	Full information	Difference
*A*	1.299*** (0.076)	0.748*** (0.078)	0.551*** (0.109)
*r*	0.109 (0.095)	0.085 (0.093)	0.0248 (0.133)
a	0.573***	0.421***	0.152***
*ρ*	−0.123	−0.092	−0.030
lnL	−64.96	−124.15	
*n*	242	330	

Note.

*** p < 0.001,

** p < 0.05,

* p < 0.1.

**Table 8:** Effect of Information Treatment on Payments to Parents

	All	Play strangers 1st	Play parents 1st
Full information (T2 & T3)	1.455*** (0.544)	2.313*** (0.640)	0.454 (0.872)
Price of giving < 1	4.154*** (0.388)	4.347*** (0.482)	3.962*** (0.620)
Price of giving > 1	−1.077*** (0.220)	−1.200*** (0.318)	−0.954*** (0.312)
Total tokens = 60	−0.394** (0.188)	−0.133 (0.185)	−0.655* (0.325)
Total tokens = 75	0.763*** (0.231)	1.135*** (0.276)	0.391 (0.363)
Total tokens = 80	0.443** (0.217)	0.875*** (0.244)	0.011 (0.346)
Total tokens = 100	1.825*** (0.244)	2.371*** (0.341)	1.280*** (0.325)
Number of observations	572	286	286
R^2^	0.4485	0.5357	0.3651

Notes.

*** p < 0.01,

** p < 0.05,

* p < 0.1.

Robust standard errors in parentheses, clustered by respondent. The unit of observation is the particular dictator game. The sample is restricted to respondents with weak preferences. The dependent variable is the amount given to parents in each game (pounds sterling). These results are robust to controlling for player characteristics (gender, age, education, student status, marital status, whether player has children) and identity of parent recipient.

## Footnotes

1 As the framing and particular wording used in dictator games influence behaviour (Hoffman *et al.*, 1996), the wording used in outlining the games was as close as possible to that of Andreoni and Miller (2002). Online Appendix C provides all instructions given to subjects.

2 Communication has been shown to generate cooperative behaviour, particularly when players are averse to guilt, that is, ‘decision makers experience guilt if they believe they let others down’ (Charness and Dufwenberg, 2006, p. 1580).

3 We refer the reader to Crawford and De Rock (2014), who provide a comprehensive review of the literature on revealed preference methods, including how GARP can be tested empirically.

4 A Monte Carlo simulation was used to estimate the relative area numerically. Relative areas were calculated by randomly drawing 50,000 choices from each budget using a uniform distribution across the entire budget and testing for whether each random choice set satisfies GARP. *a* is the proportion of these random choices that satisfy rationality. We estimate *a* = 0.051 for our budget environment.

5 This test is more demanding than one that treats giving to parents and strangers as separate goods as now 22 choices must be consistent with one another. We estimate *a* = 0.0026 when giving to the two recipients are treated as the same good.

6 Due to the limited size of our choice set and the fact that checking WARP is sufficient for checking GARP in our two-good setting, it was not necessary to implement the procedure of Smeulders *et al.* (2013) for computational reasons.

7 Quasi-linear preferences imply transferable utility. Perfect substitute preferences are quasi-linear in all goods. As we do not test directly for quasi-linear preferences, we can only reject the assumption of transferable utility for subjects with strong preferences that are not perfect substitutes. We leave testing for quasi-linearity utility functions (beyond the special case of perfect substitutes) to future work.

8 A limitation of this study is that we do not observe behaviour outside the laboratory. We did not follow up with subjects in order to ensure them that their decisions were made privately and would remain anonymous. For complete details on experimental procedures, see online Appendix B.

9 Results were not sensitive to other distance measures, for example squared deviation and absolute deviation.

10 Additional analysis in online Appendix B suggests that the assumption of homothetic or Gorman Polar Form preferences cannot be rejected for the majority of these subjects, which provides good grounds for our choice of CES utility function.

11 The estimates for *a* from games with strangers are quite similar to estimates in Andreoni and Miller (2002), where *a* = 0.76 for the weakly selfish; *a* = 0.58 for those with weakly perfect substitutes preferences; and *a* = 0.65 for those with weakly Leontief preferences.

12 Further, we believe it unlikely that giving a positive amount in such laboratory experiments to parents would be a desirable way to signal love to one’s parents. In fact, past research on gift giving has generally found that ‘familial gift giving is more like primitive premarket exchange… where gifts provide social insurance-than like signaling during courtship, so the inefficiencies that are important for signaling purposes need not be present in gift giving in the family’ (Camerer, 1988, p. S198).

13 For details on the experimental procedure, see online Appendix B.

14 Results are very similar to those presented here when Treatments 2 and 3 are included separately in the regressions.

15 We also ran the same regressions on payment amounts to strangers, and the information treatment had no effect on this outcome. Though there were similar income and price effects, they were not as strong as in games with parents.

16 We also note that players are significantly less likely to give zero to parents in the full information treatment group only in the case when they first play games with strangers. The information treatment has no effect on this outcome when players first play with parents. If giving behaviour was due to signalling, then the information treatment would not differentially impact the likelihood of giving parents zero for those who played with strangers first or those who played with parents first.

17 No subjects played selflessly with strangers. However, the precise amount of individual winnings from games with strangers was unknown to players during the experiment, as they were only paid for one of the 11 budgets, chosen at random. This was not revealed until the end of the entire experiment, when games had been played with both recipients.

18 Note that all subjects receive a show-up fee of £4. As we are interested in differences across treatments, we do not take this into account in our analysis.
