# Supplementary material for: FOR LOVE OR REWARD? CHARACTERISING PREFERENCES FOR GIVING TO PARENTS IN AN EXPERIMENTAL SETTING
Source: Econ J (London). Author manuscript; Available in PMC 2017 Nov 17. (PMC5693374; doi:10.1111/ecoj.12248)
Supplement: appendix — A. Subjects’ Notes. Appendix B. Supplementary Information on Experiments and Related Analysis. Appendix C. Laboratory Materials: Instructions to Subjects and Letter to Parents. Data S1. [file NIHMS863580-supplement-appendix.pdf]

The Economic Journal, Doi: 10.1111/ecoj.12248 © 2015 The Authors. The Economic Journal published by John Wiley & Sons Ltd on behalf of Royal Economic Society. Published by John Wiley & Sons, 9600 Garsington Road, Oxford OX4 2DQ, UK and 350 Main Street, Malden, MA 02148, USA.

Technical Appendix to

FOR LOVE OR REWARD? CHARACTERISING PREFERENCES FOR GIVING TO PARENTS IN AN EXPERIMENTAL SETTING

ECONOMIC JOURNAL, doi: 10.1111/ecoj.12248

Appendix A. Subjects' Notes[1]

A.1. *Explaining Perfect Substitutes Behaviour to Parents*

1. Wow, carbon copy paper! I feel like a 1950s secretary. If you get nothing in this study, it is because I got lots. Well, a minimum of 6 GBP. Similarly, if I get nothing, it is because you get at least 12 GBP. It seemed wise to maximise our mutual profit...
2. See attached letter XD sorry if theres no cash on the card, chose the path that equaled most cash (for a randomly selected one of us) from ___, maybe with money):P) (well, on a giftcard) ___ has new sunglasses 1337 Huh? Um... ___
3. Dear Parents, all will be revealed by telephone. I used maths to get the most utility for either parties
4. Hi Mama, might be something or nothing but between us we've won a bottle of wine I hope! Xxx ___.

A.2. *Explaining Leontief Behaviour to Parents*

1. Word up! I did this experiment thing – they said you guys might get a voucher. I hope you get something good! I didn't give you zero for anything! But it's all up to chance & which option they randomly pick. LOVE YE ALL! ___ xxx
2. To Mum, Dad and ___, Thought I'd earn us a little treat ___. The experiment reminded me of a dividing Mars Bars
3. Split it to get the same amount as we would do on holiday! Xx (P.S. You can't convert a giftcard into Euros....:))
4. Hi Mum! I tried tot do them equally. They choose 1 of 11 choices I made. My maths as you know is shoddy! We should get the same amount or roughly. Thanks for childcare xxxxx

A.3. *Explaining Selfish Behaviour to Parents*

1. Dear Mummy, I would have allocated you more money but then I remembered you're probably sitting at home eating cake while I share in this experimental lab for 1 1/2 hours and now I don't feel so bad. See you in November, ___ xxx

[1] Proper names and initials were replaced by the authors with ___ in order to protect subjects' privacy and identity.

A.4. *Other Notes to Parents*

1. Enjoy, Mum X
2. Hi!
3. To market, to market to buy a . . .
4. Hi Mum. Hopefully her is a gift card for Sainsburys for you to spend. See you soon. ___ xx
5. Hi. I thought a few Sainsburies vouchers would come in handy! See you soon. Love ___ x
6. Hopefully you'll be able to get yourself a free bottle of hot lemon!
7. I have an aid here to your housing keeping. Wait until the date shown on the giftcard to avoid embarrassment
8. Hope whatever in here comes in useful. Love ___
9. Mum, Hopefully you'll get a £4 or £5 Sainsbury's voucher. Nothing serious! Love ___.
10. Hello, Hopefully you will get some spending money "Buy yourself an ICECREAM" ___ X :)
11. Hi Mum. Hopefully a little something I've won to share with you. Love ___ X
12. A present for you!
13. Dear Mum, Here is the gift card for shopping I explained to you. Love ___
14. Enjoy! :)
15. [Foreign Language]
16. Good Luck. ___ X
17. To Mum+Dad with love from ___ xx
18. I'll explain. Nothing to worry about Love you ___
19. Dear Mum, Buy yourself some proper brand pop and chocolate biscuits(i.e. Not Asda Smart Price) with generous gift. Your daughter
20. How to explain in such a very small space?! Love, ___
21. Hi Mum, This is from the experiment I told you about-I was a guinea pig! ___ xx
22. Mum, Hope this helps!
23. NA THEN.......EYUP. Courtesy of your little gift from Heaven
24. Guess which child this is...! X (I'll explain)
25. Hey mum and dad, Just thought I would be a lab mousey for the evening! Love ___ xx
26. Hi Guys, Have fun at Sainsburys! Love ___ xxxxx
27. As promised! ___
28. Hope you enjoy Love ___ xx
29. Hi Folks, hope you enjoy whatever's on here ___ xxx
30. Hello Mum! Hopefully you have a Sainsburys and this will be useful-I got it as a price taking part in co-operative experiment. Enjoy!P.S.if not useful for you then maybe ___? xxx___
31. Hope you did well out of this! Love ___ xxx
32. Hello, will call about this if it doesn't make sense-don't worry ___

Appendix B. Supplementary Information on Experiments and Related Analysis

B.1. *Recruitment Process and Study Sample*

During the recruitment process, subjects were told that this was a research study about adults' relationships with their parents; they were not told that this experiment was being conducted by economists. Throughout our recruitment process, we indicated that eligible participants required a non-co-residing biological parent living in the UK.[2] Participants were informed in advance of their sessions that all payments would be mailed in the form of gift cards to Sainsbury's, a popular supermarket chain in the UK, and that their parents would also potentially

[2] Nonetheless, two subjects had in fact been adopted. All other subjects but one had grown up with two biological parents (one subject had a biological mother only).

© 2015 The Authors.
The Economic Journal published by John Wiley & Sons Ltd on behalf of Royal Economic Society.

receive a gift card to Sainsbury's. At the end of the experiment, each subject also received £4 in cash as a show-up fee.

Subjects were initially recruited from the pool of experiment volunteers compiled by the Nuffield College Centre for Experimental Social Sciences (CESS). The centre's database included information on student status, concentrations of study and experience in past experiments. The database allowed us to exclude undergraduate students and economics majors from our sample.

We chose to depart from the usual subject pool of economics majors and undergraduate students, as we were interested in capturing aspects of relationships between adult children and their parents. Economics students may be familiar with the dictator game and undergraduate students generally rely on their parents for financial support. We wanted to ensure that there were subjects in our sample who were financially independent of their parents.

In order to recruit additional participants, we employed a number of other methods: fliers were handed out in front of Sainsbury's in central Oxford and Headington, with follow-up emails sent out to interested participants; fliers were posted in coffee shops, colleges and Sainsbury's staff lounges throughout Oxford; advertisements were posted in local and online listings; and emails were sent to staff in all departments and colleges at the University of Oxford and Oxford Brookes University. Any participants recruited through these methods registered with CESS, which facilitated scheduling sessions and ensured that participants did not participate in our experiments more than once.

As our experiments took place in Oxford, the majority of our study sample resides in the southeast region of the UK (94%). We compare our study sample to the subsample of respondents in the British Household Panel Survey (BHPS), a nationally representative survey in the UK, who reside in the southeast region of the UK. These descriptive statistics are summarised in Appendix Table B1. While the average age for this subsample is 30, the average age of our sample is slightly higher at 33. Approximately two-thirds of our sample are women. Finally, individuals in our study sample have higher education levels than those in the comparison sample of the BHPS. While 16% of the BHPS sample have a graduate or higher degree, 36% of our study sample have a graduate degree. Nearly 85% of our sample are college-educated, while only 24% of the BHPS subsample are college-educated. The extent to which our findings may be generalised to a wider population may reflect the extent to which gender and education may influence behaviour in this particular context. Note that our findings are robust to controlling for gender, education and age.

However, we do find that men are more likely to pass GARP than women, and this difference is statistically significant. Other individual characteristics do not predict the likelihood of passing GARP. These regression results are summarised below in Appendix Table B2.

Table B1

Descriptive Statistics and Comparison to British Household Panel Survey

	British Household Panel Survey	Subjects in laboratory experiments
Southeast region	14.4%	94.2%
Age	30.0	33.0
Female	53.4%	66.3%
Education		
Higher degree (MSc, PhD)	16.5%	36.4%
First degree (BA, BEd, BSc)	7.3%	48.4%
Other degree	51.3%	15.3%
None of the above	21.1%	0.0%

Notes. Data from British Household Panel Survey include the subsample of those from the southeast region of the UK, except for region variable. Sample weights are included in BHPS averages.

© 2015 The Authors.
The Economic Journal published by John Wiley & Sons Ltd on behalf of Royal Economic Society.

Table B2

Determinants of the Likelihood of Passing GARP (Logit Regressions)

	Pass GARP with strangers				Pass GARP with parents			
Play strangers 1st	−1.072**	−1.148**	−1.127**	−1.104**	−0.434	−0.530	−0.513	−0.540
	(0.499)	(0.507)	(0.504)	(0.542)	(0.513)	(0.522)	(0.521)	(0.549)
Male	0.966	1.008	0.995	1.314*	1.705**	1.808**	1.764**	1.971**
	(0.611)	(0.624)	(0.618)	(0.741)	(0.809)	(0.835)	(0.823)	(0.912)
Age<25	1.169	1.572	1.421	1.220	0.494	0.988	0.823	1.111
	(0.903)	(0.975)	(0.960)	(1.097)	(0.832)	(0.921)	(0.902)	(1.034)
Age 25–34	0.115	0.413	0.279	0.051	0.023	0.387	0.247	0.414
	(0.612)	(0.679)	(0.660)	(0.810)	(0.679)	(0.743)	(0.726)	(0.858)
Age 35–44	0.063	0.145	0.077	0.809	0.288	0.456	0.350	0.977
	(0.764)	(0.775)	(0.769)	(1.021)	(0.854)	(0.876)	(0.862)	(1.058)
College or higher	−0.249	−0.065	−0.112	−0.393	0.627	0.911	0.825	0.545
	(0.714)	(0.742)	(0.737)	(0.963)	(0.785)	(0.833)	(0.817)	(0.957)
First degree	0.261	0.300	0.271	−0.108	0.609	0.723	0.659	0.513
	(0.712)	(0.721)	(0.716)	(0.925)	(0.727)	(0.748)	(0.737)	(0.861)
No children		−0.735		−0.690		−0.916		0.290
		(0.707)		(0.921)		(0.795)		(0.978)
No bio children			−0.516				−0.690	
			(0.701)				(0.780)	
Only mother alive				−1.038				−0.226
				(0.739)				(0.827)
Parents separated, mother is recipient				−0.432				−0.933
				(0.670)				(0.653)
No. observation	190	189	189	177[a]	190	189	189	177[a]

Notes. Standard errors in parentheses. *** $p < 0.01$, ** $p < 0.05$, * $p < 0.1$. [a] Additional controls include parents' marital status.

B.2. *Experimental Procedures*

The first experimental session was a paper and pencil pilot, which was held in May 2011. All subsequent sessions were played on the computer. There were 19 sessions in all, which were held during October 2011. All but one session was held at 5:30 pm in order to facilitate participation of those working full time. Due to multiple requests on the part of potential participants, one session was held on a Friday afternoon. As this session time proved inconvenient for too many potential subjects, all remaining sessions were held in the evening.

Prior studies have found that subjects' decisions may be influenced by a lack of anonymity and confidentiality in their choices, particularly towards what might be considered pro-social behaviour (Hoffman *et al.*, 1994; Levitt and List, 2007).[3] We designed our experiment in order to ensure that subjects' anonymity and confidentiality was maintained to the greatest extent possible, so that the experimenter would not be aware of any specific decisions, and so that subjects would not be influenced by any expectations on the part of the experimenter.[4] When a subject arrived in the laboratory at the Nuffield College Centre for Experimental Social Sciences (CESS), he was asked to address a brown envelope to himself and a white envelope to his parents. If his parents lived at separate addresses, he was instructed to address the parent envelope to his

[3] Related, social pressure has been found to be another explanation for charitable giving (DellaVigna *et al.*, 2012).
[4] See below for instructions and other materials provided to subjects.

© 2015 The Authors.
The Economic Journal published by John Wiley & Sons Ltd on behalf of Royal Economic Society.

mother. Subjects held onto these envelopes throughout the session. Before entering the laboratory, the experimenter examined both envelopes to ensure that the brown envelope was addressed to the respondent to a local address, that the white envelope was addressed to a different parent address and that both addresses were in the UK (gift cards were only valid in the UK). Each subject then picked up a claim ticket from those laid out on a table facing downward by the entrance to the laboratory. On the other side of the square was a number, and subjects were instructed to sit at the computer station with this number. They were instructed not to speak to one another and to await further instructions from the experimenter.

Subjects were informed that all allocation decisions would be kept strictly confidential. The person who conducted the experiment was not involved in doling out payments and subjects were told this at the start of the experiment. In addition, the experimenter asked for one subject to volunteer to accompany the experimenter at the conclusion of the session to verify that all payments were being mailed out that day. This was done in order to assure the participants that the transfers would indeed be made, as any doubts regarding this would also potentially influence behaviour (Bolton *et al.*, 1998). Payment allocations were recorded by each respondent's claim ticket number. In a room separate from the laboratory, research assistants inputted payment amounts onto gift cards and placed them in numbered payment envelopes corresponding to each ticket number. Brown numbered payment envelopes included cards to subjects and white numbered payment envelopes included cards to parents. Letters and any additional information being sent to parents were also included in these white envelopes. The contents of these envelopes did not include the subject's name or ticket number and subjects were informed of this in order to assure them that their parents would not have any information that we could later use to match to their responses. At the end of the experiment, subjects were called individually by their claim ticket number. A research assistant gave the subject his brown payment envelope and asked that he examine the gift card and then place it in the brown envelope he addressed to himself. A second research assistant gave the subject the white payment envelope, and asked the subject to examine the contents and place them in the white envelope he addressed to his parents. If the subject was also given an opportunity to write a note, the subject placed the top copy of his note, which indicated his claim ticket number, in a large envelope marked 'NOTES,' and the bottom copy which did not have a claim ticket number, was to be placed in the white envelope addressed to the parent. The subject was asked to seal both envelopes and place them in a larger envelope marked 'MAIL'. All of these measures were taken to assure the subject of his anonymity.

B.3. *Deviations From Rationality*

In this subsection, we compute the significance of rationality violations in our sample, to check for patterns in violations that may be linked to our experimental design. For example, did violations occur for 'early' budgets if people were confused about the game or for 'late' budgets due to fatigue?

B.3.1. *Afriat efficiency*

Firstly, we compute the 'Afriat Efficiency Index', e, for individuals who fail GARP, which returns the extent to which we would have to relax each budget constraint for the restrictions associated with GARP to be satisfied.[5] Thus, $e \in (0,1]$, with e further from 1 indicative of more significant violations of rationality (Afriat, 1981).

Table B3 shows that e reflects the differences in pass rates between the two recipients, and the low pass rate for the test of the recipients pooled together. The set of games in which parents are

[5] On power indices for revealed preference tests, see Bronars (1987) and Andreoni and Harbaugh (2006).

© 2015 The Authors.
The Economic Journal published by John Wiley & Sons Ltd on behalf of Royal Economic Society.

Table B3

Afriat Efficiency Index

Recipient	Mean pass rate	e
Stranger	0.884	0.917
Parent	0.905	0.945
Pooled	0.268	0.835

the recipients achieves the highest pass rate of 90.5%, with $e = 0.945$. When strangers are the recipients, the pass rate and e are slightly lower, 88.4% and $e = 0.917$. Our subjects treat giving to the two recipients as distinct goods. When we pool all dictator games together, only 26.8% of subjects pass GARP and $e = 0.835$. This is a relatively low Afriat Efficiency Index, so that the low GARP pass rates when all games are pooled is not simply a reflection of a more difficult test; rather, preferences for giving to parents and strangers are distinctly different for most subjects.

B.3.2. *Size of Largest Rational Choice Set*

Calculating the largest number of choices over which GARP is satisfied is an alternative way to assess the severity of deviations from rationality. Behaviour can be thought of as 'more rational' the fewer the number of choices that must be dropped for the remaining set to satisfy GARP. However, there are some subtle complications to contend with when calculating this metric. The set of choices that must be dropped for the remaining set to satisfy GARP is not necessarily unique. We adapt the partitioning algorithm defined in Crawford and Pendakur (2013) to calculate the largest number of choices over which GARP is satisfied when considering giving to parents and strangers as different goods (Table B4).

We also examined whether subjects were more likely to make 'mistakes' in the budgets they saw early on when they were learning the game, or perhaps later on when they got tired. In games with strangers, people were more likely to make 'mistakes' in the first or last three budgets seen. For 17 subjects, one of the first three budgets seen caused them to fail GARP and for 14 subjects, one of the last three budgets seen did so, whereas one of the middle three budgets caused an issue for eight subjects. But in games with parents, 11 subjects made errors in the last three budgets seen, whereas eight subjects made errors in both the first three budgets and middle three budgets. Note the order of the budgets was randomised across subjects, so that these differences are not due to any particular budget. But each subject saw the budgets in the same order in both sets of games. So differences between games with parents and strangers cannot be explained by any particular budgets.

Table B4

Rational Choice Sets

Recipient	Pass rate	No. failing	Minimum budgets dropped				
			1	2	3	4	5
Stranger	0.884	22	9	6	4	1	2
Parent	0.905	18	9	5	3	1	0

© 2015 The Authors.
The Economic Journal published by John Wiley & Sons Ltd on behalf of Royal Economic Society.

B.4. *Testing the Homothetic Axiom of Revealed Preference (HARP) and Gorman Polar Form*

In footnote 9 in the main article, we note that tests of the Homothetic Axiom of Revealed Preference (HARP) and Gorman Polar Form preferences suggest that the majority of subjects with weak preferences can have their choices rationalised by preferences with linear Engel curves. We here note the tests that we performed and also how we computed the necessary optimisation error to rationalise the behaviour of those failing both tests. To establish whether a subject's choices satisfy HARP, we check for the existence of a non-empty feasible set, $\{u_i\}_{i=1,\ldots,T,}$ to the following linear programme:

$$u_i - u_j \leq u_j \mathbf{p}_j'(\mathbf{q}_i - \mathbf{q}_j). \tag{B.1}$$

The existence of a solution to this programme is necessary and sufficient for choices to be rationalised by a homothetic utility function (Varian, 1983).

To compute the optimisation error necessary to rationalise choices, we found the minimal e (to 2 decimal places) such that the following set of inequalities are satisfied:

$$u_i - u_j \leq u_j \mathbf{p}_j'(\mathbf{q}_i - e\mathbf{q}_j). \tag{B.2}$$

This is a modification of Varian's (1990) 'goodness of fit' approach for the standard utility maximisation model. As the programme is non-linear, we performed a grid search on e at the implementation stage. If choices perfectly satisfy HARP and no optimisation error is required then $e = 1$. If choices violate HARP, then choices cannot be perfectly rationalised by a homothetic utility function and $e < 1$.

To establish whether a subject's choices can be rationalised by Gorman Polar Form preferences (this weakens above to allow a non-zero intercept for the linear Engel curves), we check for the existence of a non-empty feasible set, $\{u_i\}_{i=1,\ldots,T,}$ to the following linear programme:

$$\begin{gathered} w_s - w_t \leq \beta_t \mathbf{p}_t'(\mathbf{q}_s - \mathbf{q}_t), \\ w_t = \alpha_t + \beta_t \mathbf{p}_t' \mathbf{q}_t \end{gathered} \tag{B.3}$$

and $\alpha_t = \alpha_t$ and $\beta_t = \beta_t/\delta$, when $\mathbf{p}_t = \delta \mathbf{p}_s$. The existence of a solution to this programme is necessary and sufficient for choices to be rationalised by a Gorman Polar Form preference (Cherchye *et al.*, 2011).

To compute the optimisation error necessary to rationalise choices, we found the minimal e (to 2 decimal places) such that the following set of inequalities is satisfied:

$$\begin{gathered} w_s - w_t \leq \beta_t \mathbf{p}_t'(\mathbf{q}_s - e\mathbf{q}_t) \\ w_t = \alpha_t + \beta_t \mathbf{p}_t' \mathbf{q}_t. \end{gathered} \tag{B.4}$$

This is again a modification of Varian's (1990) 'goodness of fit' approach for the standard utility maximisation model. As the programme is non-linear, we performed a grid search on e at the implementation stage.

Interestingly, we find that many weak preference subjects could have their choices rationalised by a homothetic utility function. We find that 34% of subjects with weak preferences have perfectly homothetic preferences when giving to parents compared to 23% of those when giving to strangers. This implies that 80% of those who pass GARP when playing with parents and 71% of subjects when playing with strangers, can have their choices rationalised by a homothetic utility function (as all of the strong preferences types examined are homothetic). Typically, only minimal optimisation error is required to rationalise the choices of weak preference types by a homothetic utility function (see Table B5). Thus, homotheticity of social preferences is well supported for our sample.[6] Once one allows for a non-zero intercept of the Engel curve, (as with Gorman polar form), the behaviour of the majority of those with weak preferences is perfectly

[6] We did not compute the MPI here, as adjusting the MPI for homothetic preferences was beyond the scope of this article.

© 2015 The Authors.
The Economic Journal published by John Wiley & Sons Ltd on behalf of Royal Economic Society.

Table B5

Proportion of Homothetic Preferences amongst Weak Preference Types

	HARP		Gorman Polar	
	Parent	Stranger	Parent	Stranger
Pass ($e = 1$)	0.34	0.32	0.71	0.69
	(0.066)	(0.056)	(0.063)	(0.058)
Proportion passing with optimisation error, e				
$e \geq 0.90$	0.82	0.87	1.00	1.00
$e \geq 0.80$	0.95	0.97	1.00	1.00
$e \geq 0.60$	1.00	1.00	1.00	1.00

Note. Standard errors in parentheses.

rationalisable and those requiring some optimisation error, typically required only a 0.01–0.03 level of inefficiency.

References

Afriat, S. (1981). 'On the constructability of consistent price indices between several periods simultaneously', in (A. Deaton, ed.), *Essays in Applied Demand Analysis*, Cambridge: Cambridge University Press.

Andreoni, J. and Harbaugh, W.T. (2006). 'Power indices for revealed preference tests', Working Paper, University of California, San Diego.

Bolton, G.E., Katok, E. and Zwick, R. (1998). 'Dictator game giving: rules of fairness versus acts of kindness', *International Journal of Game Theory*, vol. 27(2), pp. 269–99.

Bronars, S.G. (1987). 'The power of nonparametric tests of preference maximization', *Econometrica*, vol. 55 (3), pp. 693–698.

Cherchye, L., Crawford, I., De Rock, B. and Vermeulen, F. (2011). 'Aggregation without aggregation: nonparametric analysis of the representative consumer', http://papers.ssrn.com/sol3/papers.cfm?abstract_id=1974867 (last accessed: 14 October 2014).

Crawford, I. and Pendakur, K. (2013). 'How many types are there?', Economic Journal, vol. 123(567), pp. 77–95.

DellaVigna, S.S., List, J. and Malmendier, U. (2012). 'Testing for altruism and social pressure in charitable giving', *Quarterly Journal of Economics*, vol. 127(1), pp. 1–56.

Hoffman, E., McCabe, K., Shachat, K. and Smith, V. L. (1994). 'Preferences, property rights and anonymity in bargaining games', *Games and Economic Behavior*, vol. 7(4), pp. 346–80.

Levitt, S.D. and List, J.A. (2007). 'What do laboratory experiments measuring social preferences reveal about the real world?', *Journal of Economic Perspectives*, vol. 21(2), pp. 153–174.

Varian, H.R. (1983). 'Nonparametric tests of consumer behaviour', *Review of Economic Studies*, vol. 50(1), pp. 99–110.

© 2015 The Authors.
The Economic Journal published by John Wiley & Sons Ltd on behalf of Royal Economic Society.

Appendix C: Laboratory Materials: Instructions to Subjects and Letters to Parents

Claim Ticket Number___________

INSTRUCTIONS

Welcome

Thank you for agreeing to participate in this experiment at the Nuffield Centre for Experimental Social Sciences (CESS). The entire experiment will have two parts and should take approximately one hour. A brief survey will follow the experiment.

This is an experiment about how people make decisions. You will be paid for participating, and the amount of money you will earn depends on the decisions you make. At the end of the experiment, you will be paid privately for your decisions.

A research foundation has provided the funds for this experiment.

Your Identity

You will never be asked to reveal your identity to anyone during the course of the experiment. Your name will never be recorded by anyone. The experimenters will not be able to link you to any of your decisions. In order to keep your decisions private, *please do not reveal your choices to any other participant.*

Payment Envelopes

Before entering the lab, you received two addressed envelopes: a brown envelope addressed to you and a white envelope addressed to your parent(s). Please place these at the top of your desk. You will be asked to present them at the end of the experiment.

Claim Ticket

When you entered the lab, you received a ticket with a number on it. This is your Claim Ticket. Each participant has a different number. You may want to verify that the number on your Claim Ticket is the same as the number on the top of this page.

You will present your Claim Ticket to an assistant at the end of the experiment to receive your payment.

Please remove your Claim Ticket now and put it in a safe place with your payment envelopes.

© 2015 The Authors.
The Economic Journal published by John Wiley & Sons Ltd on behalf of Royal Economic Society.

Claim Ticket Number__________

EXPERIMENT – PART A

You are asked to make a series of choices about how to divide a set of tokens between yourself and one other subject in the room. You and the other subject will be paired randomly and you **<u>will not</u>** be told each other's identity.

As you divide the tokens, you and the other subject will each earn money. Each choice you make is similar to the following:

Example: Divide **50** tokens:
Hold _____ tokens at 10 pence each, and *Pass* _____ tokens at 20 pence each.

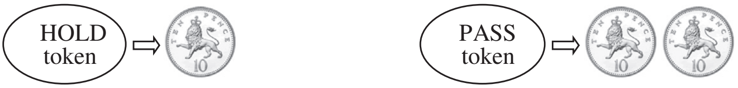

In this choice you must divide 50 tokens. You can keep all the tokens, keep some and pass some, or pass all the tokens. In this example, you will receive 10 pence for every token you hold, and the other player will receive 20 pence for every token you pass.

For example, if you hold 50 and pass 0 tokens, you will receive 50 points, or 50 × £0.10 = £5.00, and the other player will receive no points and £0. If you hold 0 tokens and pass 50, you will receive £0 and the other player will receive 50 × £0.20 = £10.00. However, you could choose any number between 0 and 50 to hold. For instance, you could choose to hold 29 tokens and pass 21. In this case you would earn 29 × £0.10 = £2.90, and the other subject would receive 21 × £0.20 = £4.20.

Here is another example:
Example: Divide **40** tokens:
Hold _____ tokens at 30 pence each, and *Pass* _____ at 10 pence each.

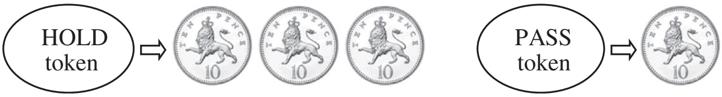

In this example every token you hold earns you £0.30, and every token you pass earns the other subject £0.10.

<u>Important Note</u>: In all cases you can choose any number to hold and any number to pass, but the number of tokens you hold plus the number of tokens you pass *must* equal the total number of tokens to divide.

Please feel free to use the scrap paper and calculator provided by the experimenter. On the screen, please click on the calculator button beside each decision question to see payment amounts based on your decisions and to assure that all of the tokens have been allocated as you would like.

© 2015 The Authors.
The Economic Journal published by John Wiley & Sons Ltd on behalf of Royal Economic Society.

Claim Ticket Number___________

EARNING MONEY IN THIS EXPERIMENT

You will be asked to make 11 allocation decisions like the examples we discussed above. We will calculate your payment as follows:

After all your decisions have been made in this part, the computer will randomly pair you with another subject in this experiment, and will select one of your decisions from this part to carry out. From this part, you will then get the tokens you allocated in the ‘hold’ portion of your decision at the indicated value, and the other subject will get the tokens you allocated on the ‘pass’ portion of your decision at the indicated value. The earnings from your decision in this part will be recorded.

Next you will be paired again with a different subject in the experiment. This time, the computer will randomly choose one of the other subject’s decisions from this part to carry out. You will earn the tokens allocated in the ‘pass’ portion from this part at the indicated values. Your earnings from this pairing will also be recorded.

The payment amounts calculated from Parts A and B will be summed up. We will send you a Sainsbury’s gift card with this total amount on the card in the envelope addressed to you. As it takes time to process payments to Sainsbury’s, please note that you will receive your card before it becomes active. Please note the activation date indicated in the card wallet. You will also receive an additional £4 show-up fee in cash.

After all the calculations have been made in Parts A and B, another experimenter who was not involved in the experiment until this time will ask you to bring up your claim ticket and will hand you your earnings envelope. This will again help to guarantee your privacy. You will present your addressed brown envelope with the opening facing upwards so that the assistant cannot see your name and address on it. You will verify that the correct payment amount has been recorded beside the number on the gift card being mailed to you, and sign a receipt for this payment. You will place your gift card in the brown payment envelope and seal it, placing it in a larger envelope with all envelopes to be mailed out that day.

A monitor chosen at the beginning of this experiment will verify that all of these payments are mailed out at the end of the session.

On the following pages are the choices we would like you to make for Part A. Please complete the form, taking the time you need to be accurate. When all subjects are done, we will instruct you on how to move on to Part B.

Thank you very much for your participation.

© 2015 The Authors.
The Economic Journal published by John Wiley & Sons Ltd on behalf of Royal Economic Society.

Claim Ticket Number___________

DECISION SHEET – PART A

Directions: Please fill in all the blanks below. Click on the calculator button to see how much you and the recipient will each be paid as a result of your decision. Feel free to make changes to your decisions until you are pleased with the payment allocations. By clicking on the calculator button, you will also be told if you have allocated more tokens than are available. Please answer all questions. Please note that once you click on the Finish button below, you will not be able to change your answers.

1. Divide **40** tokens:

*Hold*____ tokens at 10 pence each, and *Pass*____ tokens at 40 pence each.

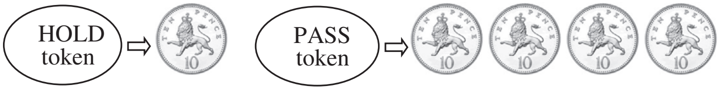

2. Divide **60** tokens:

*Hold*____tokens at 10 pence each, and *Pass*____tokens at 20 pence each.

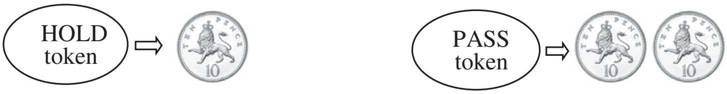

3. Divide **75** tokens:

*Hold*____tokens at 10 pence each, and *Pass*____tokens at 20 pence each.

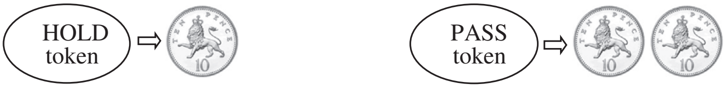

4. Divide **80** tokens:

*Hold*____ tokens at 10 pence each, and *Pass*____ tokens at 10 pence each.

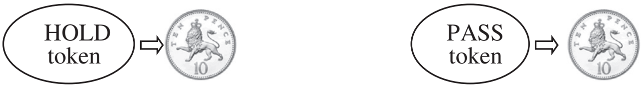

5. Divide **60** tokens:

*Hold*____ tokens at 20 pence each, and *Pass*____ tokens at 10 pence each.

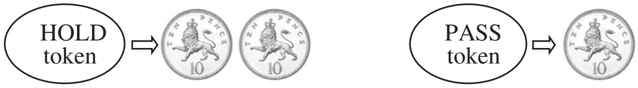

6. Divide **60** tokens:

*Hold*____ tokens at 10 pence each, and *Pass*____ tokens at 10 pence each.

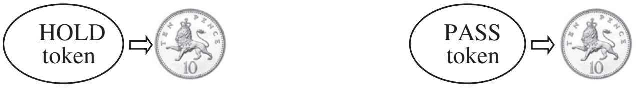

7. Divide **40** tokens:

*Hold*____tokens at 10 pence each, and *Pass*____tokens at 30 pence each.

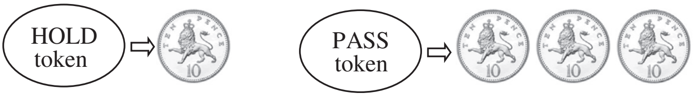

© 2015 The Authors.
The Economic Journal published by John Wiley & Sons Ltd on behalf of Royal Economic Society.

8. Divide **40** tokens:

*Hold*____tokens at 30 pence each, and *Pass*____tokens at 10 pence each.

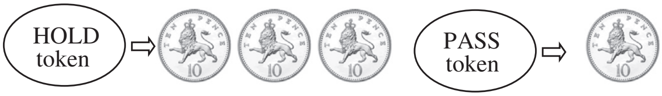

9. Divide **100** tokens:

*Hold*____ tokens at 10 pence each, and *Pass*____ tokens at 10 pence each.

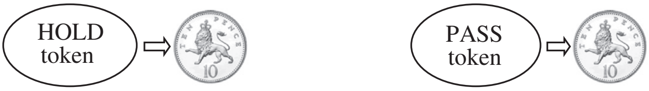

10. Divide **40** tokens:

*Hold*____ tokens at 40 pence each, and *Pass*____ tokens at 10 pence each.

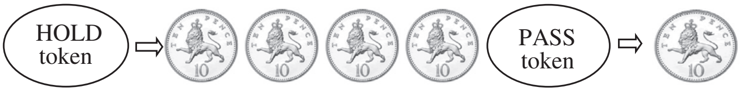

11. Divide **75** tokens:

*Hold*____tokens at 20 pence each, and *Pass*____tokens at 10 pence each.

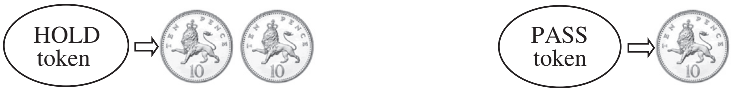

© 2015 The Authors.
The Economic Journal published by John Wiley & Sons Ltd on behalf of Royal Economic Society.

Claim Ticket Number___________

EXPERIMENT – PART B

You are asked to make a series of choices about how to divide a set of tokens between yourself and your parent(s). Please note that if both of your parents are living and live at separate addresses, we ask that you divide these tokens with your mother.

As you divide the tokens, you and your parent(s) will each earn money. Each choice you make is similar to the following:

Example: Divide **50** tokens:

Hold _____ tokens at 10 pence each, and *Pass* _____ tokens at 20 pence each.

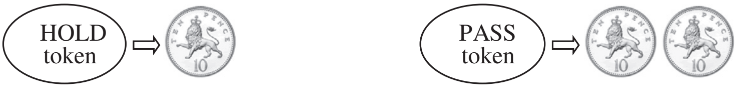

In this choice you must divide 50 tokens. You can keep all the tokens, keep some and pass some, or pass all the tokens. In this example, you will receive 10 pence for every token you hold, and your parent(s) will receive 20 pence for every token you pass.

For example, if you hold 50 and pass 0 tokens, you will receive 50 points, or 50 × £0.10 = £5.00, and your parent(s) will receive no points and £0. If you hold 0 tokens and pass 50, you will receive £0 and your parent(s) will receive 50 × £0.20 = £10.00. However, you could choose any number between 0 and 50 to hold. For instance, you could choose to hold 29 tokens and pass 21. In this case you would earn 29 × £0.10 = £2.90, and your parent(s) would receive 21 × £0.20 = £4.20.

Here is another example:

Example: Divide **40** tokens:

Hold _____ tokens at 30 pence each, and *Pass* _____ at 10 pence each.

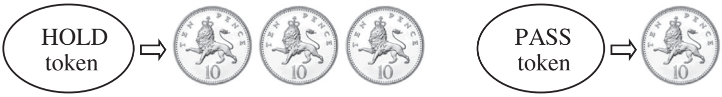

In this example every token you hold earns you £0.30, and every token you pass earns your parent (s) £0.10.

Important Note: In all cases you can choose any number to hold and any number to pass, but the number of tokens you hold plus the number of tokens you pass *must* equal the total number of tokens to divide.

Please feel free to use the scrap paper and calculator provided by the experimenter. On the screen, please click on the calculator beside each decision question to see payment amounts based on your decisions and to assure that all of the tokens have been allocated as you would like.

© 2015 The Authors.
The Economic Journal published by John Wiley & Sons Ltd on behalf of Royal Economic Society.

Claim Ticket Number___________

EARNING MONEY IN THIS EXPERIMENT

You will be asked to make 11 allocation decisions like the examples we have just discussed. We will calculate your payment as follows:

The computer will select one of your decisions to carry out. You will then get the tokens you allocated in the 'hold' portion of your decision at the indicated value, and your parent(s) will get the tokens you allocated on the 'pass' portion of your decision at the indicated value. The earnings from your decision in this part will be recorded.

The payment amounts calculated from Parts A and B will be summed up. We will send you a Sainsbury's gift card with this total amount on the card in the envelope addressed to you. As it takes time to process payments to Sainsbury's, please note that you will receive your card before it becomes active. Please note the activation date indicated in the card wallet. You will also receive an additional £4 show-up fee in cash.

The payment amount to your parents that is calculated in this part will be inputted onto the Sainsbury's gift card that will be mailed in the envelope addressed to them.

After all the calculations have been made in Parts A and B, another experimenter who was not involved in the experiment until this time will ask you to bring up your claim ticket and will hand you your earnings envelope. This will again help to guarantee your privacy. You will present your addressed brown envelope with the opening facing upwards so that the assistant cannot see your name and address on it. You will verify that the correct payment amount has been recorded beside the number on the gift card being mailed to you, and sign a receipt for this payment. You will place your gift card in the envelope and seal it, placing it in a larger envelope with all envelopes to be mailed out that day.

The payment to your parent(s) will be sent in the white envelope. You will place the gift card to your parent(s) in the white envelope addressed to them, seal it and place it in the larger envelope with all envelopes to be mailed out that day.

A monitor chosen at the beginning of this experiment will verify that all of these payments are mailed out at the end of the session.

Please note that if your parent(s) did not receive a payment, a gift card will be enclosed, but it will have no value. The card wallet will indicate that they did not receive an amount. All participants' parents will receive a letter regardless of whether or not a payment is made.

© 2015 The Authors.
The Economic Journal published by John Wiley & Sons Ltd on behalf of Royal Economic Society.

Claim Ticket Number___________

{ADDITIONAL INSTRUCTIONS FOR TREATMENTS 2 AND 3}

{The letter that will be mailed to your parent(s) is enclosed here. Please read this letter and return it to the experimenter when instructed to do so. Your parent(s) will also receive a printed copy of all of the decisions you have made in this part. This will be placed in an envelope to be collected with your payment to ensure your privacy. You may review this before placing the letter and decision sheet in the white envelope addressed to your parent(s).

For some parents, receiving this letter and payment may cause confusion and concerns. You may want to discuss this with them to alleviate their concerns. However, you do not need to tell them anything more than what has been communicated in the letter. That is entirely up to you. We have also provided information in case they would like to contact us. However, we will not divulge any more information than what has been provided here. The decision to divulge any other information is entirely and wholly left up to you. Moreover, as we will not have information to identify you, we will have no way to connect any parent who contacts us with any participant.}

{ADDITIONAL INSTRUCTIONS FOR TREATMENT 3}

{Finally, we would also like to give you an opportunity to write a personal note to your parent(s). If you wish to write a note to your parent(s), please do so on the enclosed carbon copy paper. Please keep this with your claim ticket and addressed envelopes. When you present your claim ticket, we will ask you to place the top copy in an envelope to ensure your privacy, and to place the other copy in the envelope addressed to your parents.}

On the following pages are the choices we would like you to make for Part B. Please complete the form, taking the time you need to be accurate. When all subjects are done, we will instruct you on the final part of this experiment.

Thank you very much for your participation.

© 2015 The Authors.
The Economic Journal published by John Wiley & Sons Ltd on behalf of Royal Economic Society.

Claim Ticket Number__________

DECISION SHEET – PART B

Directions: Please fill in all the blanks below. Click on the calculator button to see how much you and the recipient will each be paid as a result of your decision. Feel free to make changes to your decisions until you are pleased with the payment allocations. By clicking on the calculator button, you will also be told if you have allocated more tokens than are available. Please answer all questions. Please note that once you click on the Finish button below, you will not be able to change your answers.

1. Divide **40** tokens:

*Hold*____ tokens at 10 pence each, and *Pass*____ tokens at 40 pence each.

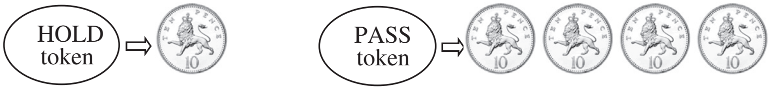

2. Divide **60** tokens:

*Hold*____tokens at 10 pence each, and *Pass*____tokens at 20 pence each.

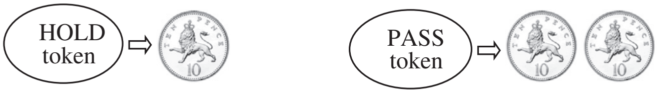

3. Divide **75** tokens:

*Hold*____tokens at 10 pence each, and *Pass*____tokens at 20 pence each.

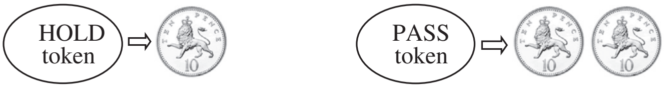

4. Divide **80** tokens:

*Hold*____ tokens at 10 pence each, and *Pass*____ tokens at 10 pence each.

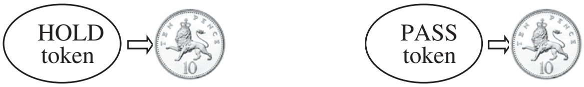

5. Divide **60** tokens:

*Hold*____ tokens at 20 pence each, and *Pass*____ tokens at 10 pence each.

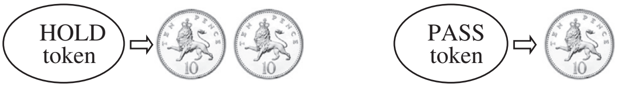

6. Divide **60** tokens:

*Hold*____ tokens at 10 pence each, and *Pass*____ tokens at 10 pence each.

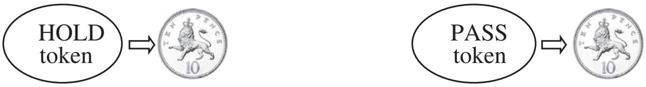

7. Divide **40** tokens:

*Hold*____tokens at 10 pence each, and *Pass*____tokens at 30 pence each.

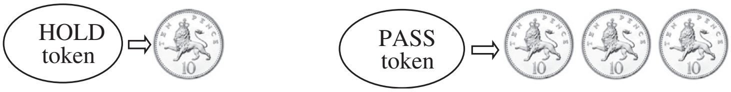

© 2015 The Authors.
The Economic Journal published by John Wiley & Sons Ltd on behalf of Royal Economic Society.

8. Divide **40** tokens:

*Hold*____tokens at 30 pence each, and *Pass*____tokens at 10 pence each.

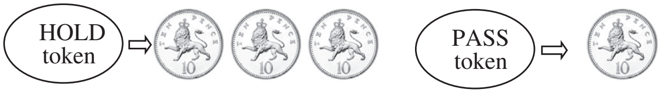

9. Divide **100** tokens:

*Hold*____ tokens at 10 pence each, and *Pass*____ tokens at 10 pence each.

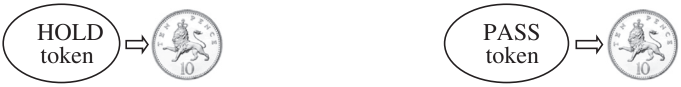

10. Divide **40** tokens:

*Hold*____ tokens at 40 pence each, and *Pass*____ tokens at 10 pence each.

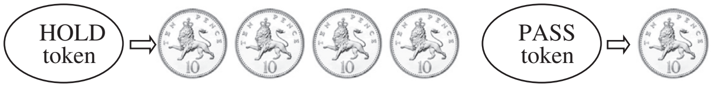

11. Divide **75** tokens:

*Hold*____tokens at 20 pence each, and *Pass*____tokens at 10 pence each.

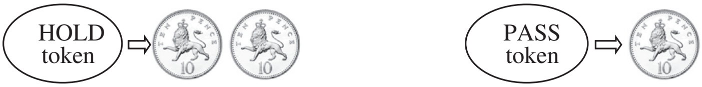

© 2015 The Authors.
The Economic Journal published by John Wiley & Sons Ltd on behalf of Royal Economic Society.

{TREATMENT 1}

Letter to Your Parent(s)

The letter that will be mailed to your parent(s) is enclosed here. Please read this letter and return it to the experimenter when instructed to do so. This will be placed in an envelope to be collected with your payment to ensure your privacy. You may review this letter again before placing it in the white envelope addressed to your parent(s).

For some parents, receiving this letter and payment may cause confusion and concerns. You may want to discuss this with them to alleviate their concerns. However, you do not need to tell them anything more than what has been communicated in the letter. That is entirely up to you. We have also provided information in case they would like to contact us. However, we will not divulge any more information than what has been provided here. The decision to divulge any other information is entirely and wholly left up to you. Moreover, as we will not have information to identify you, we will have no way to connect any parent who contacts us with any participant.

© 2015 The Authors.
The Economic Journal published by John Wiley & Sons Ltd on behalf of Royal Economic Society.

UNIVERSITY OF OXFORD

{TREATMENT 1}

Dr Maria Porter
Research Fellow
maria.porter@nuffield.ox.ac.uk
Tel. 01865 612813

Nuffield College
New Road
Oxford
OX1 1NF

5 September 2011

Dear Parent of Study Participant,

Your child has participated in a research study at the University of Oxford. The purpose of this study is to learn about how people make decisions.

As a result of his/her participation in this study, you may or may not receive a small gift. Please note that not all parents receive a gift. In order to adhere to the guidelines of this study, we must notify parents of all participants, whether or not they receive a gift.

Enclosed is a Sainsbury's gift card valued at the amount you have been allocated. If you have not been allocated a gift, the value of the enclosed gift card is zero. The value of your gift card is indicated along with the date it will be activated and available for use. Please note that you may not be able to use this card prior to this date.

In order to ensure your privacy, we have not recorded your name and address, and we will not contact you any further.

If you have any questions or concerns, please do not hesitate to contact me.

Sincerely,

Dr. Maria Porter

Nuffield College University of Oxford New Road Oxford OX1 1NF
tel 01865 612813 maria.porter@nuffield.ox.ac.uk

© 2015 The Authors.
The Economic Journal published by John Wiley & Sons Ltd on behalf of Royal Economic Society.

{TREATMENT 2}

Letter to Your Parent(s)

The letter that will be mailed to your parent(s) is enclosed here. Please read this letter and return it to the experimenter when instructed to do so. Your parent(s) will also receive a printed copy of all of the decisions you have made in this part. This will be placed in an envelope to be collected with your payment to ensure your privacy. You may review this before placing the letter and decision sheet in the white envelope addressed to your parent(s).

For some parents, receiving this letter and payment may cause confusion and concerns. You may want to discuss this with them to alleviate their concerns. However, you do not need to tell them anything more than what has been communicated in the letter. That is entirely up to you. We have also provided information in case they would like to contact us. However, we will not divulge any more information than what has been provided here. The decision to divulge any other information is entirely and wholly left up to you. Moreover, as we will not have information to identify you, we will have no way to connect any parent who contacts us with any participant.

© 2015 The Authors.
The Economic Journal published by John Wiley & Sons Ltd on behalf of Royal Economic Society.

{TREATMENT 2}

Dr Maria Porter
Research Fellow
maria.porter@nuffield.ox.ac.uk
Tel. 01865 612813

Nuffield College
New Road
Oxford
OX1 1NF

5 September 2011

Dear Parent of Study Participant,

Your child has participated in a research study at the University of Oxford. As a result of his/her participation in this study, you may or may not receive a small gift.

The purpose of this study is to learn about how people make decisions. All participants were asked to play a series of games in which they allocated a set of tokens between themselves and a parent. As they divided the tokens between themselves and you, they earned money for themselves and for you. We have enclosed instructions for these games and the decisions your child made in these games. We chose at random one of the 11 games played by your child and carried out your child's decision for that game. In the attached, we let you know how your child played this game and the outcomes of it. This determined how much money was to be paid to you and to your child.

Please note that not all parents receive a gift. In order to adhere to the guidelines of this study, we must notify parents of all participants, whether or not they receive a gift.

Enclosed is a Sainsbury's gift card valued at the amount you have been allocated. If you have not been allocated a gift, the value of the enclosed gift card is zero. The value of your gift card is indicated along with the date it will be activated and available for use. Please note that you may not be able to use this card prior to this date.

In order to ensure your privacy, we have not recorded your name and address, and we will not contact you any further.

If you have any questions or concerns, please do not hesitate to contact me.

Sincerely,

Dr. Maria Porter

Nuffield College University of Oxford New Road Oxford OX1 1NF
tel 01865 612813 maria.porter@nuffield.ox.ac.uk

© 2015 The Authors.
The Economic Journal published by John Wiley & Sons Ltd on behalf of Royal Economic Society.

UNIVERSITY OF OXFORD

HOW THE GAME WAS PLAYED

All participants were asked to play a series of games in which they allocated a set of tokens between themselves and a parent. As they divided the tokens between themselves and you, they earned money for themselves and for you.

Your child played 11 different games and allocated between 40 and 100 tokens each time. In some games, each token your child held for him or her self was worth 10 pence each. But in some games it was worth 20, 30, or 40 pence. Similarly, each token passed on to you was worth either 10, 20, 30, or 40 pence, depending on the game.

In every game, your child could choose any number to hold and any number to pass, but the number of tokens held plus the number of tokens passed must have equalled the total number of tokens to divide.

We have enclosed the decisions your child made for these 11 different games.

After your child played the 11 different games, we randomly chose one of these games to carry out. In the game that was chosen at random:

Your child divided _____ tokens:

Holding _____ tokens at ____ pence each, and Passing at _____ tokens at ____ pence each.

As a result, your child received £______ and you receive £______ .

Your payment is enclosed. Thank you for your time.

© 2015 The Authors.
The Economic Journal published by John Wiley & Sons Ltd on behalf of Royal Economic Society.

{TREATMENT 3}

Letter to Your Parent(s)

The letter that will be mailed to your parent(s) is enclosed here. Please read this letter and return it to the experimenter when instructed to do so. Your parent(s) will also receive a printed copy of all of the decisions you have made in this part. This will be placed in an envelope to be collected with your payment to ensure your privacy. You may review this before placing the letter and decision sheet in the white envelope addressed to your parent(s).

For some parents, receiving this letter and payment may cause confusion and concerns. You may want to discuss this with them to alleviate their concerns. However, you do not need to tell them anything more than what has been communicated in the letter. That is entirely up to you. We have also provided information in case they would like to contact us. However, we will not divulge any more information than what has been provided here. The decision to divulge any other information is entirely and wholly left up to you. Moreover, as we will not have information to identify you, we will have no way to connect any parent who contacts us with any participant.

Finally, we would also like to give you an opportunity to write a personal note to your parent(s). If you wish to write a note to your parent(s), please do so on the enclosed carbon copy paper. Please keep this with your claim ticket and addressed envelopes. When you present your claim ticket, we will ask you to place the top copy in an envelope to ensure your privacy, and to place the other copy in the envelope addressed to your parent(s).

© 2015 The Authors.
The Economic Journal published by John Wiley & Sons Ltd on behalf of Royal Economic Society.

{TREATMENT 3}

Dr Maria Porter
Research Fellow
maria.porter@nuffield.ox.ac.uk
Tel. 01865 612813

Nuffield College
New Road
Oxford
OX1 1NF

5 September 2011

Dear Parent of Study Participant,

Your child has participated in a research study at the University of Oxford. As a result of his/her participation in this study, you may or may not receive a small gift.

The purpose of this study is to learn about how people make decisions. All participants were asked to play a series of games in which they allocated a set of tokens between themselves and a parent. As they divided the tokens between themselves and you, they earned money for themselves and for you. We have enclosed instructions for these games and the decisions your child made in these games. We chose at random one of the 11 games played by your child and carried out your child's decision for that game. In the attached, we let you know how your child played this game and the outcomes of it. This determined how much money was to be paid to you and to your child.

Please note that not all parents receive a gift. In order to adhere to the guidelines of this study, we must notify parents of all participants, whether or not they receive a gift.

Enclosed is a Sainsbury's gift card valued at the amount you have been allocated. If you have not been allocated a gift, the value of the enclosed gift card is zero. The value of your gift card is indicated along with the date it will be activated and available for use. Please note that you may not be able to use this card prior to this date.

Your child was also given the option of writing you a note. If your child chose to do so, we have enclosed it here along with your gift card.

In order to ensure your privacy, we have not recorded your name and address, and we will not contact you any further.

If you have any questions or concerns, please do not hesitate to contact me.

Sincerely,

Dr. Maria Porter

Nuffield College University of Oxford New Road Oxford OX1 1NF
tel 01865 612813 maria.porter@nuffield.ox.ac.uk

© 2015 The Authors.
The Economic Journal published by John Wiley & Sons Ltd on behalf of Royal Economic Society.

UNIVERSITY OF OXFORD

HOW THE GAME WAS PLAYED

All participants were asked to play a series of games in which they allocated a set of tokens between themselves and a parent. As they divided the tokens between themselves and you, they earned money for themselves and for you.

Your child played 11 different games and allocated between 40 and 100 tokens each time. In some games, each token your child held for him or her self was worth 10 pence each. But in some games it was worth 20, 30, or 40 pence. Similarly, each token passed on to you was worth either 10, 20, 30, or 40 pence, depending on the game.

In every game, your child could choose any number to hold and any number to pass, but the number of tokens held plus the number of tokens passed must have equalled the total number of tokens to divide.

We have enclosed the decisions your child made for these 11 different games.

After your child played the 11 different games, we randomly chose one of these games to carry out. In the game that was chosen at random:

Your child divided _____ tokens:

Holding _____ tokens at ____ pence each, and Passing at _____ tokens at ____ pence each.

As a result, your child received £______ and you receive £______ .

Your payment is enclosed. Thank you for your time.

© 2015 The Authors.
The Economic Journal published by John Wiley & Sons Ltd on behalf of Royal Economic Society.
